# Comparative Evaluation of Effectiveness of Standard of Care Alone and in Combination With Homoeopathic Treatment in COVID-19–Related Rhino-Orbito-Cerebral Mucormycosis (ROCM): Protocol for a Single Blind, Randomized Controlled Trial

**DOI:** 10.2196/57905

**Published:** 2025-03-19

**Authors:** Harleen Kaur, Jyoti Sachdeva, Ramesh Bawaskar, Twinkle Goyal

**Affiliations:** 1 Central Council for Research in Homeopathy New Delhi India; 2 Dr.D.P. Rastogi Central Research Institute Homeopathy Noida India; 3 Regional Research Institute for Homoeopathy Mumbai India

**Keywords:** Rhino-orbital-cerebral mucormycosis, randomized controlled trial, homoeopathy, fungus, CE-MRI PNS mucormycosis, India, medical care, mortality rate, conventional therapy, ethical, mortality, survival, recovery, homoeopathic medicines, management

## Abstract

**Background:**

Rhino-orbital-cerebral mucormycosis (ROCM) is the most common (45%-74%) mucormycosis in India. With contemporary medical care, ROCM has a mortality rate of 40%-50% and 70% of survivors are left with residual defects. Recently, several cases of mucormycosis in people with COVID-19 have been increasingly reported worldwide, from India, due to immune dysregulation caused by SARS-CoV-2. To reduce the high mortality rate and residual defect in most survivors under the guidelines of the Ministry of AYUSH, the Government of India recommended homoeopathy as an add-on therapy to maximize the effectiveness of standard treatment in conventional therapy.

**Objective:**

This study aimed to evaluate the role of existing homoeopathic treatment as an adjuvant therapy in patients with COVID-19–related ROCM and enhancing the survival of the patients hospitalized due to COVID-19 infection and to access the initial treatment response and duration required for significant or complete recovery in patients receiving adjuvant treatment.

**Methods:**

This superiority, randomized controlled clinical trial would include two parallel comparator groups A and B. Group A would be the experimental group and would receive homoeopathic treatment along with the standard line of treatment as per investigational medicinal product (IMP) and group B would be the control arm and would receive standard line of treatment as per IMP along with identical placebo. Allocation would be 1:1 through randomization. Based on the inclusion and exclusion criteria, 36 participants per arm would be screened. Participants would be assessed clinically twice a day and magnetic resonance imagery or endoscopy cum-biopsy would be assessed on days 1, 14, and 28. Laboratory investigations may vary as per demand of disease conditions.

**Results:**

In India, the COVID-19 pandemic, particularly during the second wave, resulted in a surge of mucormycosis cases among patients with COVID-19. At the time this protocol was being developed, there was a significant spike in mucormycosis cases in India, particularly in Mumbai (June 2021). However, by the time the Central Council for Research in Homoeopathy obtained the necessary approvals and ethical clearance for the study, the incidence of mucormycosis had drastically declined (September 2021). As a result, the study was not initiated and registered. The authors feel it is their ethical responsibility to share the reviewed protocol with the medical community as a reference for future work.

**Conclusions:**

This study aims to evaluate the role of existing homoeopathic medicines as an adjuvant therapy in managing COVID-19–related ROCM, potentially contributing to the use of homoeopathy as an evidence-based medical approach. The protocol can also serve as a valuable resource for clinicians and researchers addressing mucormycosis cases unrelated to COVID-19, particularly in immunocompromised patients. It would help ensure preparedness, whether or not sufficient evidence is available, in the event of a future health emergency.

**International Registered Report Identifier (IRRID):**

PRR1-10.2196/57905

## Introduction

Immune dysregulation induced by SARS-CoV-2, along with the extensive use of glucocorticoids and immunomodulatory drugs like tocilizumab, has heightened the risk of secondary bacterial and fungal infections in patients with COVID-19 [1-3]. Recently in past, India experienced a significant surge in mucormycosis cases, caused by a group of molds known as mucoromycetes, commonly found in the environment. These molds release spores that are easily aerosolized and dispersed [4]. The most frequent genera causing infections in humans include *Rhizopus* and *Mucor* species, along with others like *Apophysomyces*, *Rhizomucor*, *Cunninghamella*, *Lichtheimia*, *Cokeromyces*, and *Saksenaea* [5]. *Rhizopus oryzae* is the most prevalent species, responsible for nearly 60% of human mucormycosis cases and about 90% of the rhino-orbital-cerebral mucormycosis (ROCM) form [6]. The primary mode of infection is through the inhalation of fungal spores.

Both *Aspergillus* and *Candida* have been identified as the primary fungal pathogens involved in co-infections among patients with COVID-19 [7]. Prolonged hospitalization, with or without mechanical ventilation, is also a contributing factor to these types of fungal infections [8]. Studies suggested that COVID-19 infection is associated with the destruction of β-cell of the pancreas [9,10]. A history of corticosteroid use for treating COVID-19 was found in 76.3% of mucormycosis cases, with remdesivir use in 20.6%, and tocilizumab in 4.1%. The most commonly affected organs were the nose and sinuses (88.9%), followed by rhino-orbital involvement (56.7%), and the ROCM type (22.2%) [8]. Uncontrolled hyperglycemia and the onset of diabetic ketoacidosis are often linked to corticosteroid use. COVID-19 infection can lead to endotheliitis, endothelial damage, thrombosis, lymphopenia, and reduced CD4+ and CD8+ lymphocyte counts, which increase susceptibility to secondary or opportunistic fungal infections [8]. Mucormycetes can invade blood vessels and spread to the brain and other organs through the bloodstream, leading to disseminated infections [11]. Diagnosis of ROCM should include early radiological imaging with a magnetic resonance imaging (MRI) of the paranasal sinuses (PNSs) and the brain with contrast, and confirmation can be made through fungal staining or culture from properly collected specimens [12].

ROCM is the most common form of mucormycosis in India, accounting for 45%-74% of cases. Complications of ROCM include blindness [13] cerebral infarction, cerebral abscess, cavernous sinus thrombosis, and intracranial hemorrhages [14]. The disease progresses rapidly and has a poor prognosis if not diagnosed early. Early detection, aggressive management of the underlying condition, surgical debridement, systemic and local antifungal treatments, and hyperbaric oxygen therapy significantly improve prognosis and survival rate [15]. The standard treatment for mucormycosis involves correcting diabetic ketoacidosis or other metabolic disturbances, daily irrigation and packing of the affected orbital and paranasal areas with amphotericin B, along with intravenous amphotericin B therapy [16].

### Staging of ROCM

Stage 1 of ROCM has involvement of the nasal mucosa and subdivision 1a is limited to the middle turbinate, 1b involves inferior turbinate and ostium of the nasolacrimal duct, 1c involves nasal septum, and 1d involves bilateral nasal mucosal involvement.

Stage 2 of ROCM has involvement of paranasal sinuses and subdivision 2a involves only 1 sinus, 2b involves 2 ipsilateral sinuses, 2c involves more than 2 ipsilateral sinuses and palate or oral cavity and 2d has involvement of bilateral paranasal sinuses, the zygoma and mandible [17].

Despite all efforts in the conventional line of treatment, ROCM has a mortality rate of 40%-50% and 70% of survivors are left with residual defects [18]. To reduce the high mortality rate and residual defect in most of the survivors, Ministry of AYUSH, Government of India [19] has recommended homoeopathy as an add-on therapy to maximize the effectiveness of the standard line of treatment in conventional therapy. It is a well-established fact that homoeopathy is helpful as an adjuvant therapy along with the standard line of treatment for treating various disease conditions where conventional medicine has its own limitations [20].

In various research studies undertaken on various fungi, in vitro models showed that homoeopathy medicine could prevent the growth of the fungus. Prajapati et al [21] showed that showed that homoeopathic drugs, namely *Zingiber officinale*, *Holarrhena antidysenterica*, *Terminalia chebula*, *Allium cepa*, *Caesalpinia bonducella*, *Eucalyptus globulus*, *Ruta graveolens*, and *Thuja occidentalis* have significant antifungal activity against human pathogenic fungi *Aspergillus niger* whereas Gupta and Garg [22] revealed that homoeopathic medicines Mezereum 1000 potency showed maximum inhibition of growth of Candida albicans.

Hence, this study is intended to evaluate the role of homoeopathy as an adjuvant therapy in COVID-19–related mucormycosis and to enhance the survival of the hospitalized patients with ROCM. Due to the decline in COVID-19 cases in September 2021, the period when approvals could be sought, the study was deemed unfeasible and had to be canceled. The authors consider it their ethical responsibility to share this reviewed protocol with the professional community as a resource for further research applicable to mucormycosis cases not specifically linked to COVID-19 infection. This initiative aims to ensure preparedness with evidence, regardless of its availability if similar emergencies arise in the future. Thus, we present this protocol to the community in hopes it would be used for immunocompromised or diabetes-related mucormycosis in individuals.

### Objectives

#### Primary Objective

The primary objective is to evaluate the role of adjuvant homoeopathy in addition to the standard treatment of patients with COVID-19–related ROCM through standard parameters in respective conditions.

#### Secondary Objective

This study also aims to enhance the survival of the patients hospitalized due to COVID-19–related mucormycosis.

## Methods

### Study Design

The study is a superiority, single-blind, randomized controlled trial (RCT) with 2 groups: group A (experimental) and group B (control). Group A would receive homeopathic treatment in addition to the standard treatment for COVID-19–associated mucormycosis, as recommended by the Investigational Medicinal Product or Fungal Infections Study Forum (IMP or FISF; Multimedia Appendix 1) [23]. Group B would receive conventional treatment per the same guidelines, along with an identical placebo. Participants would be randomly assigned to either group in a 1:1 ratio using a computerized random number generator.

The trial would be planned to be conducted in a hospital setting, with informed consent obtained from patients admitted for the treatment of COVID-19–related mucormycosis. The study duration is 3 months, with an extension if needed to complete the required sample size. Patients with stage 1 or 2 COVID-19–related ROCM would be enrolled and randomized based on the trial’s inclusion criteria. Both groups would receive the same auxiliary care, with dose repetition following the same pattern in both groups.

**Figure 1:**
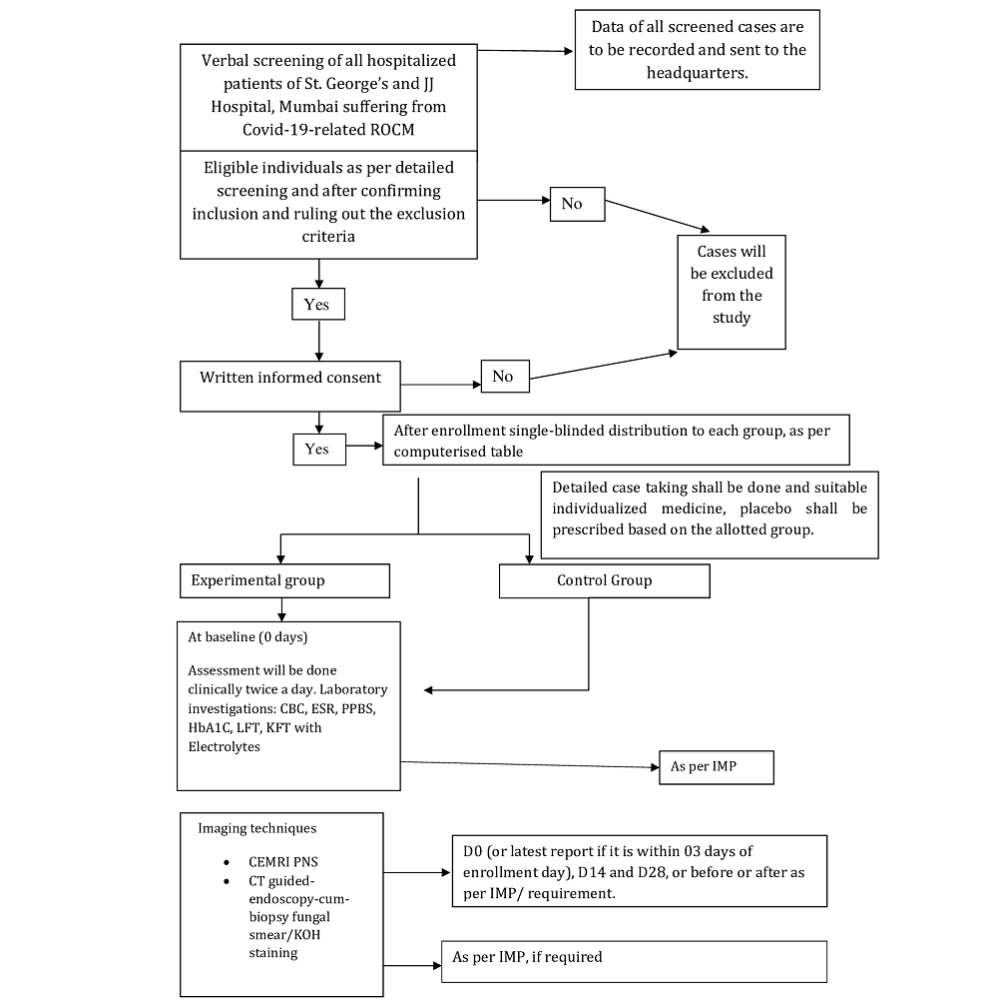
Flow diagram of study design. CEMRI PNS: Contrast-Enhanced Magnetic Resonance Imaging of Para Nasal sinus; CBC: complete blood count; CT: computed tomography; ESR: erythrocyte sedimentation rate; HbA1c: hemoglobin A1c; IMP: investigational medicinal product; KFT: kidney function test; KOH: potassium hydroxide; LFT: liver function test; PPBS: postprandial blood sugar; ROCM: rhino-orbital-cerebral mucormycosis.

Participants would be assessed clinically twice a day, and MRI or endoscopy with biopsy would be performed on days 1, 14, and 28, with variations depending on the disease progression. The study protocol adheres to the SPIRIT (Standard Protocol Items: Recommendations for Interventional Trials) 2013 guidelines, as illustrated in Figure 1.

### Study Setting and Duration

This RCT was planned for the St. George’s and JJ Hospital, Mumbai, which was the hospitals of Sir JJ Group of hospitals, and had a common Institutional Ethics Committee. An informed consent of the patients admitted in the inpatient department for treatment of COVID-19–related mucormycosis planned to be taken before inclusion. Duration of the study would be 3 months and maybe extended if the desired sample size is not achieved.

### Eligibility Criteria

The eligibility criteria are shown in Textbox 1.

**Eligibility criteria.**

**Inclusion criteria**

- Both male and female participants aged 18 years or older and confirmed cases of COVID-19–related rhino-orbital-cerebral mucormycosis (ROCM) diagnosed as stage 1 and stage 2 (Multimedia Appendix 2) by potassium hydroxide staining (KOH staining) or magnetic resonance imaging (MRI) or biopsy would be included in the study. MRI would show Mucosal thickening with T2 hypointense components at T2W images, nonenhancement of involved mucosa or soft tissue at postcontrast T1W images, Marrow edema and enhancement of adjacent bones and skull base at fat-saturated T2W and postcontrast T1W image [24]. A hyperintense lesion extending from paranasal sinus along orbital apex into intracranial structures and narrowing or slow flow in the ipsilateral internal carotid artery in the vicinity of mucor invasion seen in T2W [25]. KOH staining or microscopy and biopsy of affected area would show nonseptate or pauci-septate, ribbon-like hyphae (at least 6-16 μm wide), and vessel occlusion [26].

**Exclusion criteria**

- ROCM at stage 3 and 4 (Multimedia Appendix 2) presenting with bony erosion, cerebral vascular invasion, invasion into the cranium, orbital apex or cribriform plate of the ethmoid bone, cavernous sinus (II-VI cranial nerve palsies) and diagnosed cases of pulmonary, cutaneous, gastrointestinal, advanced disseminated mucormycosis, pregnant and lactating women, cases where progression to death is inevitable and imminent within the next 24 hours according to the clinical team irrespective of the provision of treatments would be excluded from the study.

### Intervention

#### Preparation of Study Intervention and Placebo Materials

Homoeopathic medicines to be used in the trial are known pharmacopeial preparations. As no new remedy is proposed to be investigated, there is no potential risk due to intervention. Intervention medicines would be administered orally. The medicine would be repeated depending on the potency and complaints of the patient in accordance with the principles of homoeopathy. Once the improvement sets in, placebo would be continued till the main medicine continues to act. The placebo (30 CH) would be identically prepared using only purified water, and pharmaceutical-grade ethanol, without glycerin or any medicinal active component.

#### Details of Intervention

The intervention would be a homoeopathic medicine which would be given as pills medicated with an ultradiluted homeopathic remedy. As a basic rule, increased dilution is proportionate to increased therapeutic power and no side effects. Owing to the nanoparticle nature of the drugs particles in serial dilutions above the Avogadro constant [27], homoeopathic intervention does not have any safety issue since inception. As the medicines used for this trial are already in use for decades and therefore, practical safety of medicines is well-established, this trial would be phase 3 trial and further validate the efficacy of these medicines, specifically in ROCM.

Through patient information sheet, patient would be duly informed about equal chances of receiving placebo or homoeopathic medicine, depending on the group she or he’s allotted, and then consent would be taken. Furthermore, patients would be getting standard care of conventional medicine in both groups, and, therefore, would not be at risk. A detailed history and examination would be gathered from the already available information of the patient in the admission records at hospital. The patients randomized into experimental group would be interrogated by the homoeopathic physician only for the additional information required from homoeopathy perspective.

Daily recording of the administered dose would be recorded and also captured in the case record form developed by the homoeopathy team, which would be having content other than, and in addition to the standard form, which would be filled at the hospital. If there is any change in dosage or prescription of medications during the study period, that would be mentioned with reason in the prescription chart. If these medicines are stopped, that too would be recorded with reason.

The patients assigned to the placebo group would be given placebo in the form of similar looking homoeopathic pills of size 30, dispensed with the 30% V/V dispensing alcohol. The prescribed medicine would be changed if no change in the next scheduled laboratory investigations or patient reports to be clinically unwell, even after at least 3 doses of the prescribed medicine.

In either arm, no patient would be devoid of conventional management as per IMP at any given point of time. Till the end point of the study in hospitalized patients, if required to enquire about well-being of the patient, phone call may be done in both groups and it would be considered as a phone follow-up.

FISF recommendations on Treatment of COVID-19–associated mucormycosis [23] (Multimedia Appendix 1) would be followed for regime of medication under standard care of intervention, which includes 3-6 weeks of amphotericin B therapy, followed by consolidation therapy for 3-6 months, strict control of hyperglycemia, steroids for lifesaving purposes in patients with COVID-19; abuse or misuse or untimely use to be avoided, judicious immunomodulating drugs, surgical debridement of the infected tissues, and regular monitoring through clinical, radio-imaging, and microbiological assessment.

#### For Homeopathic Intervention

As the condition is immunocompromised, very acute and progressive in nature, the homoeopathic medicines would be recommended in low potency and in frequent repetition. Medicine will be repeated in the following instances (Textbox 2).

**Condition where medicine would be repeated.**

**Repetition of medicine:**

- If improvement stops, repeat the same medicine in the same potency.
- If no further amelioration occurs even after repeating the medicine in the same potency or improvement lasts for a very short period, give higher potency of the same medicine.
- If there is too short relief of symptoms (lasting for a few hours and diminishing further on subsequent repetition of the doses), reassess the case and observe.
- If the medicine remains appropriate after reassessment, it should be administered in a next higher potency.
- If no further amelioration occurs, reassess the case and prescribe the indicated remedy.
- If aggravation is quick, short, and strong with rapid improvement of the patient, discontinue the medicine and prescribe placebo.
- If the appearance of new symptoms is observed, and if the symptoms are not of a serious nature, wait till the new symptoms pass off. Then select another indicated remedy after reassessing the case.
- In case of serious symptoms, change the medicine and check for safety end point.

**Withdrawal of a trial participant:**

- If the patient worsens, and mucormycosis expands beyond the range of inclusion criteria.
- If the patient is in need of ventilator support.
- If the participant is unwilling to continue or turns noncompliant.

**Outcome Measures**

#### Primary Outcome

The following changes would be observed in MRI contrast:

1. Mucosal thickening with reduction in Transverse Relaxation time T2 hypointense components at T2-weighted (T2W) images.
2. Infiltration of mucosa/soft tissue at postcontrast T1-weighted (T1W) images.
3. Marrow edema and enhancement of adjacent bones and skull base at fat-saturated T2W and postcontrast T1W image.
4. Hyperintense lesion extending from paranasal sinus along orbital apex into intracranial structures and narrowing or slow flow in the ipsilateral internal carotid artery in the vicinity of mucor invasion seen in T2W.
5. Staging of Code Mucor as per Guidelines for the Diagnosis, Staging and Management of ROCM in the setting of COVID-19 pandemic [17]. The absence of stage 1 would be considered as cured and progression from stage 1 to stage 2 or stage 2 to stage 3 or 4 would be considered as worse outcome.

#### Secondary Outcomes

The median survival rate of the patients would be assessed in both the groups during hospitalization.

#### Participant Timeline

Each patient would be followed up for 28 days. Participant would be followed up every 4 hours, or sooner, as per the case and would be asked a few questions as per case recording form, before commencing the treatment. Participants would be requested to be available on phone for follow up by the doctor on day 14 and day 28, in case they are discharged from the hospital before day 14 of the illness.

The following variables would be clinically assessed in every follow up:

1. Nasal stuffiness
2. Nasal discharge
3. Foul smell
4. Epistaxis
5. Facial pain
6. Facial edema
7. Dental pain
8. Malaise
9. Fever

### Sample Size

Based on the computation of sample size, literature, and expert opinions and assuming a type 1 error of 5% and type 2 error of 20% with a power of 80%, it was estimated that there would be 36 participants each in the intervention and control group, with a total of 72 participants. This could be escalated based on attrition due to loss in follow-up. If this loss to follow-up is considered at 25%, a total of 45 participants have to be enrolled in each group, with a total sample of 90 for the study.

### Randomization and Blinding

The study participants were planned to be recruited from St. George’s and JJ Hospital, Mumbai. The recruitment would be based on the inclusion criteria. The participants would be allocated to either intervention or placebo groups as per the randomization sequence generated by using GraphPad (GraphPad Software) by Dotmatics (Insightful Science) software. Coded or prenumbered identical containers would be used for allocation concealment. The principal investigator (PI) at Central Council for Research in Homoeopathy (CCRH) headquarters would be involved in the sequence generation process and allocation concealment procedure. The PI would label the interventions as per the randomization codes, which would be handed over to the site PIs. The site PIs would enroll participants and randomly assign participants to interventions. The only trial participants interventions would be blinded. To ensure the quality of blinding the packaging for both the interventions and placebo would be identical in odor, color, size, and taste. The unblinding would be done by the PI at CCRH headquarters at the end of the study. If severe adverse events would be reported by the participants during the trial, unblinding may be performed per Data and Safety Monitoring Board (DSMB) or ethical committee recommendations. The reason for urgent unblinding should be well-documented.

### Screening and Enrollment

All the participants would be voluntarily invited to the study and then required to sign an informed consent form containing information on the regulatory authorities and the related procedures, including laboratory investigations and subsequent randomization after enrollment. Enrollment of the participants would be based on inclusion criteria. The hospital must maintain a log register that records all the details of the screened participants and the reasons for exclusion. After enrollment the participants would be randomized and allocated to the intervention or the placebo arm as per the generated randomization sequence using GraphPad by Dotmatics software.

### Data Collection Methods

Considering the critical importance of COVID-19–related ROCM, data from the trial participants would be collected extensively. The following documents below would be developed and retained.

1. Case record form of COVID-19–related ROCM.
2. A database that stores the above information for each patient with the subsequent observations by the physician at every visit.
3. Reports of all laboratory investigations to help physicians understand efficacy of diagnostic tools or interventions.
4. All data pertaining to homoeopathy, like case taking, repertorization chart, and prescription decisions would be maintained.

### Assessment and Data Analysis

Potassium hydroxide staining and Contrast-Enhanced Magnetic Resonance Imaging (CEMRI PNS) would be used for diagnosis and staging of ROCM. However, for assessment of the progress of the condition, following parameters would be evaluated on days 14 and 28:

1. CEMRI PNS impression
2. Code Mucor staging
3. Clinical assessment
4. Laboratory parameters

The participants would be assessed clinically twice a day and the laboratory parameters complete blood count, erythrocyte sedimentation rate, fasting blood sugar, postprandial blood sugar, hemoglobin A_1c_ levels, liver function test, and kidney function test with electrolytes would be assessed on days 0, 14, and 28. These tests would be repeated as many times as required as per Institutional Management Protocol, which is essentially based on FISF guidelines. However, the assessments would be done only on the fixed days, as mentioned above.

CEMRI PNS would also be done on day 1 (or latest report if it is within 03 days of enrolment day), day 14, and day 28, or before or after as per IMP or FISF requirement Multimedia Appendix 3.

### Follow-Ups

Each patient would be followed up for 28 days. Participants would be followed up daily as long as they are hospitalized; in case of discharge before this follow up period, they would be followed up telephonically on day 14 and day 28.

The following variables would be clinically assessed in every follow-up:

1. Nasal stuffiness
2. Nasal discharge
3. Foul smell
4. Epistaxis
5. Facial pain
6. Facial edema
7. Dental pain
8. Malaise
9. Fever

The case would be said to be completed when there is either recovery of the participant as per the outcome parameters, followed by discharge from the hospital, or has advanced in the form of advanced disseminated mucormycosis or death of the patient.

### Data Analysis

The study center would send a weekly report as per weekly report proforma on case recruitment to headquarters either by fax or email every week. The DSMB would assess the research data regularly. The conclusion report on completion of the study (after 3 months) is to be submitted to Headquarters as per concluding report proforma.

### Withdrawal Criteria

The participant would be withdrawn from the study if the condition of the participants worsens and the mucormycosis expands beyond the inclusion criteria (safety end point), participants require ventilator support, it suggests a severe respiratory condition, possibly due to complications from mucormycosis or other underlying health issues, or participants become unwilling to continue or become noncompliant in the RCT and request the site PI to withdraw them for the RCT would be withdrawn from the study. Every participant can withdraw from the trial anytime for any reason and without prejudice.

### End Point

A case would be considered complete when the participant either recovers based on the defined outcome parameters and is discharged from the hospital within or before 28 days. Safety end points include advanced disseminated mucormycosis and patient death.

### Statistical Analysis

Data collected would be compiled on to a Microsoft Excel worksheet and would be subjected to statistical analysis using an appropriate package like SPSS (IBM Corp) software. Descriptive statistics like frequency and percentage of categorical data, mean (SD) of numerical data in each group or subgroup would be depicted. Frequency and percentage of various categories in each group or subgroup would be compared using chi-square test. Normality of numerical data would be checked using Shapiro-Wilk test or Kolmogorov-Smirnov test. Depending on the normality of data, statistical tests would be determined. For a numerical continuous data following a normal distribution, inter group comparison (2 groups) would be done using *t* test, else a nonparametric substitute like the Mann-Whitney *U* test would be used. Intra group comparisons for a numerical continuous data following a normal distribution would be carried out using paired *t* test (for 2 observations) or repeated measures ANOVA for >2 observations, else a nonparametric substitute like Wilcoxon signed-rank test (for 2 observations) or the Friedman test for >2 observations would be used. Frequency and percentage of various responses in each time interval would be compared using the chi-square test or McNemar test. Having set the α error at 5%, β error at 20%, and power at 80%, *P*<.05 would be considered statistically significant (Multimedia Appendices 4, 5, and 6).

### Data Monitoring and Management

#### Project Monitoring and Reviews

Record of each enrolled case is also to be electronically maintained and this electronically maintained record is to be sent to headquarters through email to the study coordinator at CCRH headquarters as soon the case is enrolled for verification of the case record with annexures. Concerned authority shall do on-site monitoring at 1-month interval till completion of the study.

#### Quality Control

A centralized workshop would be organized for the investigators (to be involved in specific study) to ensure standardization and quality control. A periodical review would be conducted at all the sites for quality assurance. A random subset of records from each site would be evaluated for quality control. Investigators would be asked to bring all medical records for selected subjects to the data analysis workshop. Information in the medical records would be compared with the data on the case report form to assess completeness and accuracy of reported data.

#### Harms

Homoeopathic medicines which shall be used in the trial are known pharmacopeial preparations. As no new remedy is proposed to be investigated, there is no potential risk due to intervention.

### Auditing and Inspecting

The study center would send a weekly report as per weekly report proforma on case recruitment to headquarters either by fax or email every week of the month. The DSMB would assess the research data regularly. The conclusion report on completion of the study (after 3 months) is to be submitted to hqrs. as per concluding report proforma.

### Ethical Considerations

#### Protocol Development

This is carried out in consultation with experts of St. George’s and JJ Hospital, Mumbai and then subsequently reviewed by Dr V Shankar Kumar, Consultant ENT Surgeon Apollo Spectra Hospitals, Chennai and Dr Sushil Kabra, Department of Pediatrics, AIIMS New Delhi before it was submitted to ethics committee.

#### Human Subject Ethics Review Approvals or Exemptions

The study protocol shall be in accordance with the latest revision of the Helsinki Declaration on human experimentation and Good Clinical Practices India. Although medicines proposed to be used during the study are known homoeopathic pharmacopeial preparations, necessary clearance of the Ethical Committee shall be obtained before undertaking the study. The Scientific Advisory Board and the Central Ethics Committee of the CCRH have approved the study protocol and the amendments (1-29/2021-22/CCRH/Tech/Post-covid Mucormycosis/887; July 15, 2021). The investigator must meet the study requirements as specified in the protocol. Protocol amendments are possible only in exceptional cases (eg, where the health or well-being of the participant is affected) and only after authorization by the Scientific Advisory Committee (SAC) of the Council. If there is any modification in the protocol, an addendum of the same shall be circulated to all the investigators after due approval of SAC and EC. The investigator shall update the protocol by attaching the addendum of the amendment to the already circulated protocol. In case of any administrative or technical amendments, which do not affect the health of the participant, agreement of all the concerned shall be made. These changes shall also be justified in writing and all those concerned are to be informed.

#### Informed Consent

Individuals aged 18 years or older would be considered eligible for consent. The site PI would obtain voluntary consent from the participants before the screening test after explaining the study. The participants would be provided with the participant information sheet describing the study details and would voluntarily sign the consent form if they agree to take the intervention.

#### Privacy and Confidentiality

The investigator would inform the participants that all trials results recorded would be treated in strict confidence. During documentation and analysis, the participants would only be identified by their subject code and unique identifier number. The confidentiality of the participants’ personal data would be maintained as per data protection regulations.

#### Compensation Details

CCRH would provide insurance cover in the trial for the said study duration, according to the terms finalized under clinical trial cover with the identified insurance firm.

#### Protocol Violation

If the patient stops taking the intervention medication on their own, this would be considered as protocol violation. So, these participants would not be considered for per protocol analysis.

#### Declaration of Interest

No conflict of competing interest of the PI or site PI, or any other member of the investigation team.

#### Access to Data

The access to data would be restricted for analysis and interpretation only by CCRH and other collaborating organization, as and when involved. For all purposes, the data would remain strictly confidential, and anonymity would be assured through the best possible means.

#### Ancillary and Posttrial Care

The participant would be provided with all ancillary care during the study and other ancillary techniques. Besides, she/he would be provided with regular outpatient department care, even after the study is over.

#### Dissemination Policy

The investigators would communicate trial results to participants, health care professionals, the public, and other relevant groups (through publication, reporting in results databases, or other data sharing arrangements) after completion of the study. However, no information based on unjustified claims, or the findings of interim analysis would be communicated in any form.

## Results

India faced a significant impact from the COVID-19 pandemic, with 44,587,307 confirmed cases and 528,629 deaths reported as of January 17, 2023, according to the World Health Organization [28]. The second wave placed tremendous strain on the nation’s health care system, resulting in severe shortages of drugs, vaccines, ventilators, and oxygen. During this period, there was a notable increase in mucormycosis cases among patients with COVID-19, leading the government to classify it as a notifiable disease under the Epidemic Diseases Act of 1897 on May 20, 2021 [29]. To address the high mortality rate and long-term complications experienced by many survivors, the Ministry of AYUSH, Government of India, recommended homoeopathy as an adjunct therapy to enhance the effectiveness of standard conventional treatments. The CCRH developed a protocol to incorporate homoeopathy as an additional therapy for treating COVID-19–associated mucormycosis. However, when this protocol was created, there was a significant number of mucormycosis cases in India, particularly in Mumbai (June 2021). By the time CCRH obtained all necessary approvals and ethical clearance for the study, the incidence had decreased dramatically (September 2021), resulting in the study not being initiated or registered. The authors believe it is their ethical duty to share this reviewed protocol with the medical community as a reference for future research.

## Discussion

### Principal Findings

This study was conceived during the exponential increase in Mucormycosis cases in India since March 2021. This opportunistic infection was being observed in patients with severe COVID-19. Globally, the prevalence of mucormycosis varied from 0.005 to 1.7 per million population in 2019-2020. Its prevalence in India was 0.14 per 1000 population. In May 2021, the cases peaked to 11,717 cases of mucormycosis across 18 states of India [24]. ROCM is extremely fatal with mortality rates ranging from 85%-93%, despite the best treatment in immunocompromised patients [30]. Patients with poorly controlled diabetes mellitus are the primary group at risk for developing ROCM. Other recognized risk factors worldwide include diabetes-associated ketoacidosis, hematological malignancies, solid organ or bone marrow transplants, surgeries, trauma, neutropenia, protein-calorie malnutrition, autoimmune diseases, chronic kidney disease or renal failure, HIV infection, deferoxamine therapy, and corticosteroid therapy [31]. ROCM typically starts in the paranasal sinuses and then spreads to adjacent structures, such as the orbit and neurocranium. Most reported cases are from Iran (26%), India (22%), China (17%), and the United States (15%), regions where diabetes prevalence is significantly increasing [32]. Chavda et al [33] state that mucormycosis affects the nose, eyes, and brain and is a potentially fatal intrusive fungal infection that frequently affects immunodeficient individuals.

In conventional treatment, surgical and pharmaceutical interventions are key to treat mucormycosis, thus necessitating a multidisciplinary approach team of otolaryngology, ophthalmology, neurosurgery, and infectious disease specialists in a facility setting. Intravenous antifungal pharmacotherapy is the first line of treatment, amphotericin B deoxycholate and posaconazole or isavuconazole being the 2 antifungal agents recommended for the primary therapy of mucormycosis [34]. However, the scope of treatment in limited in this condition, and resulting side effects are known. Chakraborty et al [34] have reported side effects from liposomal amphotericin B, which include fever, chills, nausea, vomiting, loss of appetite, headache, and some serious side effects which include swelling or pain at injection site, muscle or joint pain, unusual tiredness, weakness, muscle cramping and signs of kidney problems (such as a change in the amount of urine or painful urination) and so on after infusion of the drug, which, in turn, causes hindrance to continue the required conventional treatment [17]. Sachdeva et al [35] states that hepatotoxicity and nephrotoxicity are well-known serious side effects of intravenous Amphotericin B and posaconazole treatment. Pal et al [36] reports palatal, facial soft tissue necrosis, loss of vision, and invasion of soft tissues of the infratemporal fossa, orbit or palate through neurovascular structures as complications of ROCM.

Due to limited scope of conventional medicine, side effects that are serious or interfere in the treatment, advance complications and poor prognosis with high mortality rate, the authors felt a need of exploring the usefulness of alternative system of medicine to complement the standard line of treatment for COVID-19–related ROCM. The authors have had a successful experience of providing adjunct homoeopathic treatment in severe cases of COVID-19 before planning this RCT, which, is now reported [37]. Planning this RCT, thus, seemed to be a step in the right direction.

The evidence for similar studies in the past in homoeopathy sector is limited. The literature was, therefore, looked up for role of homoeopathy in fungal studies, instead. A study on dermatophytosis and its management by homoeopathic medicines reports successful reduction in the NRS and DLQI scores, thus suggesting it as a feasible integrative treatment option for tinea infections [38]. An in vitro study on antifungal activity of homoeopathic medicines against plant fungus *Aspergillus niger* observed that homoeopathic medicines with various potencies (6C to CM) had a significant antifungal activity, compared with the controls, which is bavistin (Carbendazim) and ethanol (Dispensing alcohol 90%) [39]. A case series by Roy et al [40] reports positive effect of homoeopathic medicines against dermatophytosis. Furthermore, a pilot study by Sherr et al [41] reported that homoeopathic medicine Bacillinum had a potential to improve long term tinea status and reduces the chances to relapse as compared with the standard treatment for tinea and homoeopathy can be used as an alternative to standard treatment care. Other in-vitro studies have also reported homoeopathy medicines to have worked effectively against the growth of different forms of fungus [21,22].

There was, however, no study reported so far to assess the role of homoeopathic medicines in mucormycosis. Hence, to the authors’ knowledge, this protocol was a first with the aim to evaluate the efficacy of homoeopathic medicines as an adjuvant in the treatment of COVID-19–related ROCM. However, with the decline in COVID-19 cases in September 2021, which was the time when the approvals could be sought, the study had to be called off due to nonfeasibility reasons. The authors feel this as their ethical duty to share this reviewed protocol with the profession as a reckoner or source of further work for the clinicians or scientists alike, in a more generalized manner, which is to say, in the cases of mucormycosis not particularly resulting from COVID-19 infection. This would help in being prepared with the evidence, or the lack of it, should such an emergency situation arise in future. We, thus, share this protocol with the community with the hope that it may be adopted for mucormycosis cases that are generally seen in immunocompromised individuals.

### Conclusion

This study aimed to evaluate the role of existing homoeopathic medicines as an adjuvant therapy in managing COVID-19–related ROCM, potentially contributing to the use of homoeopathy as an evidence-based medical approach. The protocol can also serve as a valuable resource for clinicians and researchers addressing mucormycosis cases unrelated to COVID-19 infection, particularly in immunocompromised patients. It would help ensure preparedness, whether or not sufficient evidence is available, in the event of a future health emergency. The authors have tried to adopt universally acceptable, standardized parameters for determining the improvement, with secondary parameters intended for evaluating the survival rate of the hospitalized patients besides other parameters that have been selected for assessing initial treatment response through laboratory and imaging techniques. A planned outcome of the study includes comparing the time required for significant or complete recovery between patients receiving adjuvant treatment and those in the control group. The authors welcome future studies based on this protocol and would be pleased to see their work advanced by fellow professionals.

## Appendix

Multimedia Appendix 1 Treatment for COVID-19-associated mucormycosis recommended by the FISF.

Multimedia Appendix 2 Staging of rhino-orbital-cerebral mucormycosis.

Multimedia Appendix 3 SPIRIT flow diagram.

Multimedia Appendix 4 Dummy table for Baseline.

Multimedia Appendix 5 Dummy table for follow ups.

Multimedia Appendix 6 Dummy table for Survival time.

Multimedia Appendix 7 Original Protocol.

## Abbreviations

CCRH: Central Council for Research in Homoeopathy
CEMRI PNS: Contrast-Enhanced Magnetic Resonance Imaging of Para Nasal sinus
DSMB: Data and Safety Monitoring Board
FISF: Fungal Infection Study Forum
IMP: investigational medicinal product
MRI: magnetic resonance imaging
PI: principal investigator
PNS: paranasal sinus
RCT: randomized controlled trial
ROCM: rhino-orbital-cerebral mucormycosis
SAC: Scientific Advisory Committee
SPIRIT: Standard Protocol Items: Recommendations for Interventional Trials
T1W: T1-weighted
T2W: T2-weighted

## Data Availability

No data are associated with the protocol as the study could not be initiated because mucormycosis cases started declining.

## References

[1] Kumar G, Adams A, Hererra M, Rojas ER, Singh V, Sakhuja A, Meersman M, Dalton D, Kethireddy S, Nanchal R, Guddati AK (2021). Predictors and outcomes of healthcare-associated infections in COVID-19 patients. Int J Infect Dis.

[2] Kimmig LM, Wu D, Gold M, Pettit NN, Pitrak D, Mueller J, Husain AN, Mutlu EA, Mutlu GM (2020). IL-6 inhibition in critically Ill COVID-19 patients is associated with increased secondary infections. Front Med (Lausanne).

[3] Garg D, Muthu V, Sehgal IS, Ramachandran R, Kaur H, Bhalla A, Puri GD, Chakrabarti A, Agarwal R (2021). Coronavirus disease (Covid-19) associated mucormycosis (CAM): case report and systematic review of literature. Mycopathologia.

[4] Richardson M (2009). The ecology of the Zygomycetes and its impact on environmental exposure. Clin Microbiol Infect.

[5] Roden MM, Zaoutis TE, Buchanan WL, Knudsen TA, Sarkisova TA, Schaufele RL, Sein M, Sein T, Chiou CC, Chu JH, Kontoyiannis DP, Walsh TJ (2005). Epidemiology and outcome of zygomycosis: a review of 929 reported cases. Clin Infect Dis.

[6] Sugar AM, Mandell GL, Bennett JE, Dolin R (2000). Mandell, Douglas, and Bennett's Principles and Practice of Infectious Diseases.

[7] Song G, Liang G, Liu W (2020). Fungal co-infections associated with global COVID-19 pandemic: a clinical and diagnostic perspective from China. Mycopathologia.

[8] Singh AK, Singh R, Joshi SR, Misra A (2021). Mucormycosis in COVID-19: a systematic review of cases reported worldwide and in India. Diabetes Metab Syndr.

[9] Müller JA, Groß R, Conzelmann C, Krüger J, Merle U, Steinhart J, Weil T, Koepke L, Bozzo CP, Read C, Fois G, Eiseler T, Gehrmann J, van Vuuren J, Wessbecher IM, Frick M, Costa IG, Breunig M, Grüner B, Peters L, Schuster M, Liebau S, Seufferlein T, Stenger S, Stenzinger A, MacDonald PE, Kirchhoff F, Sparrer KMJ, Walther P, Lickert H, Barth TFE, Wagner M, Münch J, Heller Sa, Kleger A (2021). SARS-CoV-2 infects and replicates in cells of the human endocrine and exocrine pancreas. Nat Metab.

[10] Tang X, Uhl S, Zhang T, Xue D, Li B, Vandana JJ, Acklin JA, Bonnycastle LL, Narisu N, Erdos MR, Bram Y, Chandar V, Chong ACN, Lacko LA, Min Z, Lim JK, Borczuk AC, Xiang J, Naji A, Collins FS, Evans T, Liu C, tenOever BR, Schwartz RE, Chen S (2021). SARS-CoV-2 infection induces beta cell transdifferentiation. Cell Metab.

[11] Hartnett KP, Jackson BR, Perkins KM, Glowicz J, Kerins JL, Black SR, Lockhart SR, Christensen BE, Beer KD (2019). A guide to investigating suspected outbreaks of mucormycosis in healthcare. J Fungi (Basel).

[12] (2021). Technical guideline on diagnosis and treatment of COVID-19 associated mucormycosis (CAM). COVID-10 Outbreak and Infection Control Cell, Dept of Health & Family Welfare, Government of Kerala.

[13] Simmons JH, Zeitler PS, Fenton LZ, Abzug MJ, Fiallo-Scharer RV, Klingensmith GJ (2005). Rhinocerebral mucormycosis complicated by internal carotid artery thrombosis in a pediatric patient with type 1 diabetes mellitus: a case report and review of the literature. Pediatr Diabetes.

[14] Koc Z, Koc F, Yerdelen D, Ozdogu H (2007). Rhino-orbital-cerebral mucormycosis with different cerebral involvements: infarct, hemorrhage, and ophthalmoplegia. Int J Neurosci.

[15] Mohamed MS, Abdel-Motaleb HY, Mobarak FA (2015). Management of rhino-orbital mucormycosis. Saudi Med J.

[16] Kohn R, Hepler R (1985). Management of limited rhino-orbital mucormycosis without exenteration. Ophthalmology.

[17] Honavar SG (2021). Code Mucor: guidelines for the diagnosis, staging and management of rhino-orbito-cerebral mucormycosis in the setting of COVID-19. Indian J Ophthalmol.

[18] Warwar RE, Bullock JD (1998). Rhino-orbital-cerebral mucormycosis: a review. Orbit.

[19] Information for homoeopathy practitioners for symptomatic management of suspected and diagnosed cases of mucormycosis. Ministry of Ayush Govt.

[20] Rostock M, Naumann J, Guethlin C, Guenther L, Bartsch HH, Walach H (2011). Classical homeopathy in the treatment of cancer patients--a prospective observational study of two independent cohorts. BMC Cancer.

[21] Prajapati S, Sharma M, Kumar A, Gupta P, Dwivedi B, Arya B, Arya R, Nayak D (2019). Antimicrobial activity of different homoeopathic drugs and their potencies against 'Aspergillus niger' In vitro. Indian J Res Homoeopathy.

[22] Gupta G, Garg K (1995). Effect of homoeopathic drugs against fungi isolated from human patients. Asian Homoeopathic Journal.

[23] Covid-19 associated mucormycosis. National Centre for Disease Control, Directorate General of Health Services, Government of India.

[24] Sekaran A, Patil N, Sabhapandit S, Sistla SK, Reddy DN (2022). Rhino-orbito-cerebral mucormycosis: an epidemic in a pandemic. IJID Reg.

[25] Lone P, Wani N, Jehangir M (2015). Rhino-orbito-cerebral mucormycosis: Magnetic resonance imaging. Indian J Otol.

[26] Management protocol for mucormycosis. All India Institute of Medical Sciences.

[27] Bell IR, Schwartz GE, Boyer NN, Koithan M, Brooks AJ (2013). Advances in integrative nanomedicine for improving infectious disease treatment in public health. Eur J Integr Med.

[28] WHO coronavirus (COVID-19) dashboard. World Health Organization.

[29] (2023). Make Mucormycosis a notifiable disease under epidemic act, urges Centre. Hindustan Times.

[30] Kaushal D, Rajan N, Soni K, Sharma A, Choudhury B, Yadav T, Khera P, Gupta P, Kaur N, Goyal A (2022). Reducing mortality in mucormycosis of the head and neck in diabetic patients: a CARE case series. Eur Ann Otorhinolaryngol Head Neck Dis.

[31] Dam P, Cardoso MH, Mandal S, Franco OL, Sağıroğlu P, Polat OA, Kokoglu K, Mondal R, Mandal AK, Ocsoy I (2023). Surge of mucormycosis during the COVID-19 pandemic. Travel Med Infect Dis.

[32] Beiglboeck F, Theofilou N, Fuchs M, Wiesli MG, Leiggener C, Igelbrink S, Augello M (2022). Managing mucormycosis in diabetic patients: a case report with critical review of the literature. Oral Dis.

[33] Chavda VP, Apostolopoulos V (2021). Mucormycosis - an opportunistic infection in the aged immunocompromised individual: a reason for concern in COVID-19. Maturitas.

[34] Chakraborty M, Rani P, Gupta N, Saxena N (2022). A pre-post interventional study for evaluating the usefulness of homoeopathic medicine nux vomica to combat the side effects of liposomal amphotericin B injection in the cases of mucormycosis. Homœopathic Links.

[35] Sachdeva K, Saji TA (2022). Adverse drug reaction of antifungals in the management of black fungus: a tertiary care centre experience. Indian J Otolaryngol Head Neck Surg.

[36] Pal P, Singh B, Singla S, Kaur R (2022). Mucormycosis in COVID-19 pandemic and its neurovascular spread. Eur Arch Otorhinolaryngol.

[37] Kaur H, Bawaskar R, Khobragade A, Kalra D, Packiam V, Khan MY, Kaur T, Sharma M, Verma NK, Kaushik S, Khurana A (2023). Randomised controlled trial to compare efficacy of standard care alone and in combination with homoeopathic treatment of moderate/severe COVID-19 cases. PLoS One.

[38] Dixit AK, Javed D, Srivastava A, Bala R, Giri N (2024). Homeopathic medicines in the management of dermatophytosis (Tinea Infections): a clinico-epidemiological study with pre-post comparison design. Homeopathy.

[39] Peerzada P, Aswani A, Kathade A, Jadhav B, Kunchiraman N, Shinde H (2018). In-vitro studies for anti-fungal activity of homoeopathic medicines against plant fungus aspergillus niger. International Journal of Research and Analytical Reviews Oct-Dec.

[40] Roy PS, Das S, Goswamifficacy of individualized homeopathic treatment in the management of Dermatophytosis; A case Series (2021). Efficacy of individualized homeopathic treatment in the management of dermatophytosis; a case series. Int. J. AYUSH CaRe.

[41] Sherr J, Davy J, Saghai Z, Quirk T, Fibert P (2022). The comparative effectiveness of the homeopathic medicine Bacillinum for ringworm (tinea): a pilot feasibility study. European Journal of Integrative Medicine.

