# Supplementary figures and images for: Comparative Evaluation of Effectiveness of Standard of Care Alone and in Combination With Homoeopathic Treatment in COVID-19–Related Rhino-Orbito-Cerebral Mucormycosis (ROCM): Protocol for a Single Blind, Randomized Controlled Trial
Source: JMIR Res Protoc. 2025 Mar 19;14:e57905. doi: 10.2196/57905 (PMC11966070; doi:10.2196/57905)

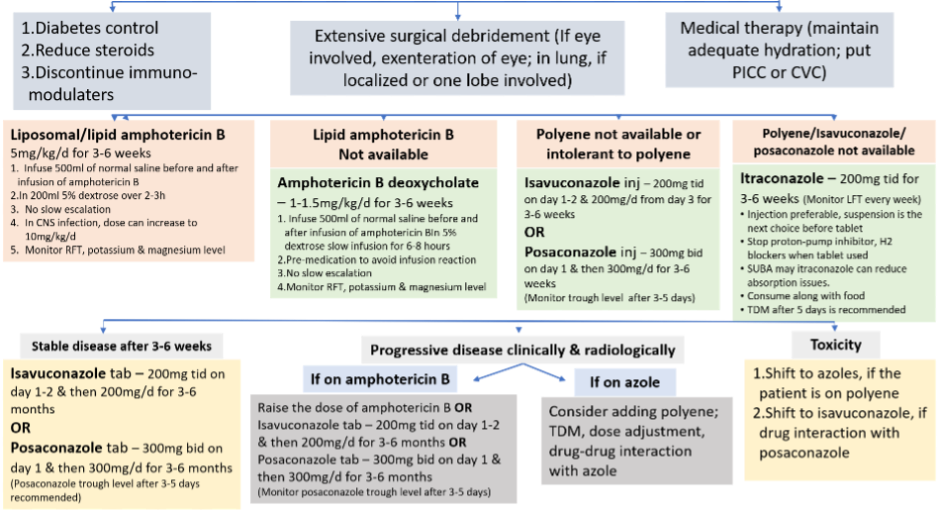

Supplement: Multimedia Appendix 1 [file resprot_v14i1e57905_app1.png]
