# Supplementary material for: Comparative Evaluation of Effectiveness of Standard of Care Alone and in Combination With Homoeopathic Treatment in COVID-19–Related Rhino-Orbito-Cerebral Mucormycosis (ROCM): Protocol for a Single Blind, Randomized Controlled Trial
Source: JMIR Res Protoc. 2025 Mar 19;14:e57905. doi: 10.2196/57905 (PMC11966070; doi:10.2196/57905)
Supplement: Multimedia Appendix 2 [file resprot_v14i1e57905_app2.docx]

**Staging of Rhino-Orbito-Cerebral Mucormycosis (ROCM)**

Source: Honavar SG. Code Mucor: Guidelines for the Diagnosis, Staging and Management of Rhino-Orbito-Cerebral Mucormycosis in the Setting of COVID-19. Indian J Ophthalmol. 2021 Jun;69(6):1361-1365. Doi: 10.4103/ijo.IJO_1165_21. PMID: 34011699.

| ***Staging of Rhino-orbito-Cerebral Mucomycosis*** | ***Symptoms*** | ***Signs*** |
| --- | --- | --- |
| **Stage:1 Involvement of the Nasal mucosa**  **1a**: Limited to the middle turbinate  **1b**: involvement of the inferior turbinate or ostium of the nasolaciacrimal duct  **1c**: involvement of the nasal septum  **1d**: Bilateral nasal mucosal involvement | Nasal Stuffiness, nasal discharge, foul smell, epistaxis | Foul-smelling sticky mucoid or haemorrhagic nasal discharge, nasal mucosal, inflammation, erythema, Violaceous or blue discoloration, pale ulcer, anaesthesia, ischemia, eschar |
| **Stage:2 Involvement of Paranasal Sinuses**  **2a**: One sinus  **2b**: Two ipsilateral sinuses  **3c**: >Two ipsilateral sinuses and/or palate/oral cavity  **3d**: Bilateral paranasal sinus involvement or involvement of the zygoma or mandible | Symptoms in stage 1+ facial pain, facial edema, dental pain, systemic symptoms (malaise fever) | Signs stage 1+ unilateral or bilateral localized or diffuse facial edema, edema localized over the sinuses, localized sinus tenderness |
| **Stage:3 Involvement of the Orbit**  **3a**: Nasolacrimal duct, medial orbit, vision unaffected  **3b**: Diffuse orbital involvement (.1 quadrant or >2 structures), vision unaffected  **3c**: Central retinal artery or ophthalmic artery occlusion or superior ophthalmic vein thrombosis; involvement of the superior orbital fissure, inferior orbital fissure, orbital apex, loss of vision | Symptoms in stages 1 and 2 + pain in the eyes, proptosis, ptosis, diplopia, loss of vision, infraorbital and facial V1 V2 nerve anesthesia | Signs in stages 1 and 2+ conjunctival chemoses, isolated ocular motility restriction ptosis, Proptosis, infraorbital nerve anesthesia, central retinal artery occlusion, features of ophthalmic vein thrombosis. V1 and V2 nerve anesthesia, and features of III , IV and VI nerve palsy indicating orbital apex/superior orbital fissure involvement. |
| **Stage:4 Involvement of the CNS**  **4a:** Focal or partial cavernous sinus involvement and/or involvement of the cribriform plate  **4b:** Diffuse cavernous sinus involvement and/or cavernous sinus thrombosis  **4c:** Involvement beyond the cavernous sinus, involvement of the skull base, internal carotid artery occlusion, brain infraction  **4d:** multifocal or diffuse CNS disease | Symptoms stages 1 and 3+ bilateral proptosis, paralysis, altered consciousness, focal seizures | Signs in Stages 1-3 (same features overlap with Stage 3) + V1 and V2 nerve anesthesia ptosis, and features of III, IV and VI nerve palsy indicate cavernous sinus involvement. Bilaterally of these signs with contralateral orbital edema with no clinic-radiological evidence of paranasal sinus or orbital involvement on the contralateral side indicate cavernous sinus thrombosis. Hemiparesis, altered consciousness and focal seizure indicate brain invasion and infraction. |
