# Supplementary material for: Comparative Evaluation of Effectiveness of Standard of Care Alone and in Combination With Homoeopathic Treatment in COVID-19–Related Rhino-Orbito-Cerebral Mucormycosis (ROCM): Protocol for a Single Blind, Randomized Controlled Trial
Source: JMIR Res Protoc. 2025 Mar 19;14:e57905. doi: 10.2196/57905 (PMC11966070; doi:10.2196/57905)
Supplement: Multimedia Appendix 3 [file resprot_v14i1e57905_app3.doc]

**Timeline Diagram**

|  | **Enrolment** | **Allocation** | **Close-out** | | |
| --- | --- | --- | --- | --- | --- |
| **TIMEPOINT**** |  | **0** | ***Day0*** | ***Day14*** | ***Day 28*** |
| **ENROLMENT:** |  |  |  |  |  |
| **Eligibility screen** | X |  |  |  |  |
| **Informed consent** | X |  |  |  |  |
| ***Baseline Characteristics*** | X |  |  |  |  |
| ***Blinding Randomization*** | X |  |  |  |  |
| **Allocation** |  | X |  |  |  |
| **INTERVENTIONS:** |  |  |  |  |  |
| ***Group A (homoeopathic intervention + standard line of treatment A)*** |  | X |  |  |  |
| ***Group B (conventional treatment + placebo)*** |  | X |  |  |  |
| **ASSESSMENTS:** |  |  |  |  |  |
| ***CT guided-endoscopy-cum-biopsy Fungal smear/KOH staining*** | X |  |  |  |  |
| ***CEMRI PNS*** | X |  | X | X | X |
| ***Code Mucor staging*** | X |  | X | X | X |
| ***Clinical assessment*** | X |  | X | X | X |
| - ***Laboratory parameters***   ***(CBC, ESR, FBS, PPBS, HbA1c, LFT, KFT with electrolytes)*** | X |  | X | X | X |
