# Supplementary material for: Comparative Evaluation of Effectiveness of Standard of Care Alone and in Combination With Homoeopathic Treatment in COVID-19–Related Rhino-Orbito-Cerebral Mucormycosis (ROCM): Protocol for a Single Blind, Randomized Controlled Trial
Source: JMIR Res Protoc. 2025 Mar 19;14:e57905. doi: 10.2196/57905 (PMC11966070; doi:10.2196/57905)
Supplement: Multimedia Appendix 4 [file resprot_v14i1e57905_app4.docx]

Baseline

| Variables | Group A (homoeopathic intervention + standard line of treatment) n(%) | Group B (conventional treatment + placebo)n(%) | P Value |
| --- | --- | --- | --- |
| Age ( Mean ± SD ) |  |  |  |
| Gender (%)  Male  Female |  |  |  |
| Association with COVID‑19  n(%)  No COVID‑19  Definite COVID‑19 Suspected COVID‑19 |  |  |  |
| Comorbiditiesn (%)  Diabetes mellitus  Hypertension  Coronary artery disease  Others |  |  |  |
| Immunocompromised |  |  |  |
| History of oxygen therapy |  |  |  |
| History of steroid use |  |  |  |
| Staging of ROCM (Code Mucor Guidelines)n (%)  1a  1b  1c  1d  2a  2b  2c  2d |  |  |  |
| KOH staining |  |  |  |
| Nasal stuffiness |  |  |  |
| Nasal discharge |  |  |  |
| Foul smell |  |  |  |
| Epistaxis |  |  |  |
| Facial pain |  |  |  |
| Facial edema |  |  |  |
| Dental pain |  |  |  |
| Malaise |  |  |  |
| Fever |  |  |  |

*Continuous data: Mean ± SD will be measure with independent t-test.

*Categorical data: % will be measure by Chi-squared test. Missing value will dealt with ITT.
