# Supplementary material for: Comparative Evaluation of Effectiveness of Standard of Care Alone and in Combination With Homoeopathic Treatment in COVID-19–Related Rhino-Orbito-Cerebral Mucormycosis (ROCM): Protocol for a Single Blind, Randomized Controlled Trial
Source: JMIR Res Protoc. 2025 Mar 19;14:e57905. doi: 10.2196/57905 (PMC11966070; doi:10.2196/57905)
Supplement: Multimedia Appendix 5 [file resprot_v14i1e57905_app5.docx]

Follow up on Day 14 and 28

| **Variables** | **Baseline** | | **Day14** | | **Day28** | | P Value |
| --- | --- | --- | --- | --- | --- | --- | --- |
| No. of patients (n) | Group A (homoeopathic intervention + standard line of treatment) | Group B (conventional treatment + placebo) | Group A (homoeopathic intervention + standard line of treatment) | Group B (conventional treatment + placebo) | Group A (homoeopathic intervention + standard line of treatment) | Group B (conventional treatment + placebo) |  |
| Staging of ROCM (Code Mucor Guidelines)  1a  1b  1c  1d  2a  2b  2c  2d |  |  |  |  |  |  |  |
| Nasal stuffiness |  |  |  |  |  |  |  |
| Nasal discharge |  |  |  |  |  |  |  |
| Foul smell |  |  |  |  |  |  |  |
| Epistaxis |  |  |  |  |  |  |  |
| Facial pain |  |  |  |  |  |  |  |
| Facial edema |  |  |  |  |  |  |  |
| Dental pain |  |  |  |  |  |  |  |
| Malaise |  |  |  |  |  |  |  |
| Fever |  |  |  |  |  |  |  |

*Continuousdata: Mean ± SD will be measure with repeated ANNOVA.

*Categorical data: % will be measure by Chi-squared test. Missing value will dealt with ITT.
