# Supplementary material for: Comparative Evaluation of Effectiveness of Standard of Care Alone and in Combination With Homoeopathic Treatment in COVID-19–Related Rhino-Orbito-Cerebral Mucormycosis (ROCM): Protocol for a Single Blind, Randomized Controlled Trial
Source: JMIR Res Protoc. 2025 Mar 19;14:e57905. doi: 10.2196/57905 (PMC11966070; doi:10.2196/57905)
Supplement: Multimedia Appendix 6 [file resprot_v14i1e57905_app6.docx]

Dummy Table for measure Survival time

|  | Mean Survival Time (Mean ± SD) | CI | Median Survival Time (IQR) | CI |
| --- | --- | --- | --- | --- |
| Group A |  |  |  |  |
| Group B |  |  |  |  |
