# Supplementary material for: Comparative Evaluation of Effectiveness of Standard of Care Alone and in Combination With Homoeopathic Treatment in COVID-19–Related Rhino-Orbito-Cerebral Mucormycosis (ROCM): Protocol for a Single Blind, Randomized Controlled Trial
Source: JMIR Res Protoc. 2025 Mar 19;14:e57905. doi: 10.2196/57905 (PMC11966070; doi:10.2196/57905)
Supplement: Multimedia Appendix 7 [file resprot_v14i1e57905_app7.docx]

**Comparative evaluation of effectiveness of standard of care alone and in combination with homoeopathic treatment in COVID-19 related Rhino-orbito-cerebral Mucormycosis (ROCM): A single blind, Randomized Controlled trial**

| **Title** | Comparative evaluation of effectiveness of standard of care alone and in combination with homoeopathic treatment in COVID-19 related Rhino-orbito-cerebral Mucormycosis (ROCM): A single blind, Randomized Controlled trial |
| --- | --- |
| Short Title | *Role of adjuvant homoeopathy in COVID related ROCM* |
| Methodology/Design | Randomised control, single blind, parallel group trial |
| Study Duration | 3 months |
| Study Centre(s) | **St. George’s and JJ Hospital, Mumbai** /Any other |
| Introduction | Recently, several cases of mucormycosis in people with COVID19 have been increasingly reported world-wide, in particular from India. The primary reason that appears to be facilitating Mucorales spores to germinate in people with COVID-19 is an ideal environment of low oxygen (hypoxia), high glucose (diabetes, new-onset hyperglycemia, steroid-induced hyperglycemia), acidic medium (metabolic acidosis, diabetic ketoacidosis [DKA]), high iron levels (increased ferritins) and decreased phagocytic activity of white blood cells (WBC) due to immunosuppression (SARS-CoV-2 mediated, steroid-mediated or background comorbidities) coupled with several other shared risk factors including prolonged hospitalization with or without mechanical ventilators.^[[1]](#endnote-1)^ Use of Homoeopathy as adjuvant to Standard Treatment has helped faster and better recovery in previous fungal infection. This knowledge may be put to use, and further validated in the treatment COVID related rhino-orbito-cerebral mucormycosis. The details are given in introduction section. |
| **Objectives** | **Primary Objective**   - To evaluate the role of adjuvant homoeopathy in treatment of covid-related ROCM patients through standard parameters in respective conditions   **Secondary Objective**   - To enhance the survival of the patients hospitalized due to covid-related mucormycosis |
| **Patients and Methods** | Patients hospitalised for treatment of covid-related ROCM will be provided Homoeopathy as adjuvant treatment with standard treatment care, after informed consent in one arm, and standard treatment care alone in the other. |
| **Number of Subjects (Sample Size)** | 72 (36 each arm); explained in Section 3.7) |
| **Inclusion Criteria** | - Stage 1 and 2 (Staging document annexed) of ROCM - Age > 18 years - All genders - Fungal smear analysis through KOH staining/ microscopy of affected area showing presence of mucormycetes (to save precious time), followed by following exploratory imaging techniques:   - CEMRI (for ruling out Stages 3 and 4)   - CT-guided endoscopy-cum-biopsy (for detailed histopathology study) |
| **Exclusion Criteria** | - ROCM cases in stages 3 and 4(Staging document annexed) presenting with bony erosion, cerebral vascular invasion, invasion into the cranium, orbital apex or cribriform plate of the ethmoid bone, cavernous sinus (II-VI cranial nerve palsies) will be excluded. - Diagnosed cases of Pulmonary ,Cutaneous, Gastrointestinal ,Advanced, disseminated mucormycosis will be excluded. - Pregnant and lactating women. - In the opinion of the clinical team, progression to death is imminent and inevitable within the next 24 hours, irrespective of the provision of treatments. |
| **Follow-up period** | - Patient will be assessed clinically twice a day; MRI/endoscopy-cum-biopsy will be assessed on day 1, 14 and 28. |
| **Outcomes** | **Primary Outcome**   1. Change in condition as seen in standard imaging or other diagnostic techniques:  - MRI contrast in ROCM cases   **Secondary Outcomes**   1. To assess difference in the survival of the patients in both the groups during hospitalization. |
| **Endpoint** | - Recovery as per the outcome parameters, followed by discharge from hospital - Advanced, disseminated mucormycosis - Death of the patient |
| **Study Product, Dose, Route** | - Individualized homoeopathic medicine in appropriate dose, orally, four times daily; or more frequently in severe cases. |
| Placebo | Placebo group patients will receive identical placebo (globules moistened with dispensing alcohol) as an add on to standard protocol treatment. Dose repetition will be a in similar pattern to medicine group. |
| Duration of administration | Maximum 28 days (depending on the patient’s admission duration) |
| Statistical Methodology | All primary efficacy analyses will be conducted in the Intention-to-treat (ITT) population; thus, all randomized patients should be included in the primary analysis. |
| Funding Agency | Central Council for Research in Homoeopathy, Ministry of AYUSH, Govt. of India |
| Budget | Rs. 75 lakhs approx. |
| Study Team | CCRH  Mentors   - Dr. Anil Khurana - Dr. Praveen Oberai, CCRH, HQ.   PI: Dr. Harleen Kaur; Research Officer/Scientist I, CCRH, Delhi  Co-I: Dr. Ramesh Bawaskar; Research Officer/ Scientist 3, Regional Research Institute (H), Mumbai  Co-I: Dr.Jyoti, RA(H), CCRH, HQ. |

# Introduction

## Background

The pandemic coronavirus disease 2019 (COVID-19) continues to be a significant problem worldwide. In the absence of an effective vaccine or antiviral therapy, supportive care plays a vital role in the management of COVID-19. Glucocorticoids and probably remdesivir are the only drugs proven to be beneficial in COVID-19. Unfortunately, the widespread use of glucocorticoids can lead to secondary bacterial or fungal infections. The immune dysregulation caused by the virus and the use of concurrent immunomodulatory drugs such as tocilizumab also increased the risk of infections in COVID-19 patients.^[[2]](#endnote-2),^^[[3]](#endnote-3),^^[[4]](#endnote-4)^

Mucormycosis is caused by a group of molds called mucormycetes, which are found in the environment and release spores that are easily aerosolized and dispersed^[[5]](#endnote-5)^ . The most common genera that cause infections in humans are Rhizopus and Mucor species, but others include Apophysomyces, Rhizomucor, Cunninghamella, Lichtheimia, Cokeromyces, and Saksenaea^[[6]](#endnote-6)^. The Rhizopus Oryzae is most common type and responsible for nearly 60% of mucormycosis cases in humans and also accounts Journal Pre-proof for 90% of the Rhino-orbital-cerebral (ROCM) form^[[7]](#endnote-7)^ . Mode of contamination occurs through the inhalation of fungal spores.

Both Aspergillosis and Candida have been reported as the main fungal pathogens for co-infection in people with COVID-19^[[8]](#endnote-8)^ . Recently, several cases of mucormycosis in people with COVID19 have been increasingly reported world-wide, in particular from India. The primary reason that appears to be facilitating Mucorales spores to germinate in people with COVID-19 is an ideal environment of low oxygen (hypoxia), high glucose (diabetes, new-onset hyperglycemia, steroid-induced hyperglycemia), acidic medium (metabolic acidosis, diabetic ketoacidosis [DKA]), high iron levels (increased ferritins) and decreased phagocytic activity of white blood cells (WBC) due to immunosuppression (SARS-CoV-2 mediated, steroid-mediated or background comorbidities) coupled with several other shared risk factors including prolonged hospitalization with or without mechanical ventilators.^[[9]](#endnote-9)^ One of the systematic review showed that mucormycosis was predominantly seen in males (78.9%), both in people who were active (59.4%) or recovered (40.6%) from COVID-19. Hyperglycemia at presentation (due to pre-existing DM or new-onset hyperglycemia or new-onset diabetes or diabetic ketoacidosis [DKA]) was the single most important risk factor observed in majority of cases (83.3%) of mucormycosis in people with COVID-19, followed by cancer (3.0%).Studies suggested that covid-19 is associated with destruction of β-cell of the pancreas^[[10]](#endnote-10)^, ^[[11]](#endnote-11)^. History of corticosteroid intake for the treatment of COVID-19 was present in 76.3% of cases, followed by remdesivir (20.6%) and tocilizumab (4.1%). Commonest organ involved with mucormycosis was nose and sinus (88.9%), followed by rhino-orbital (56.7%) and ROCM type (22.2%). ^[[12]](#endnote-12)^ Uncontrolled hyperglycemia and precipitation of Diabetic Ketoacidosis is often observed due to corticosteroid intake. Low pH due to acidosis is a fertile media for mucor spores to germinate.

Moreover, steroid use reduces the phagocytic activity of WBC (both first line and second line defense mechanism), causes impairment of bronchoalveolar macrophages migration, ingestion, and phagolysosome fusion, making a diabetic patient exceptionally vulnerable to mucormycosis. COVID-19 often causes endothelialitis, endothelial damage, thrombosis, lymphopenia, and reduction in CD4+ and CD8+ level and thus predisposes to secondary or opportunistic fungal infection.^[[13]](#endnote-13)^

Mucormycosis can affect nearly any part of the body, with rhinocerebral and pulmonary infections caused by inhalation of spores, and cutaneous infections caused by spores entering the skin. Gastrointestinal mucormycosis can occur when contaminated foods or products are introduced to the GI tract. Mucormycetes can infiltrate blood vessels and spread to the brain and other organs through the bloodstream, resulting in disseminated infections.^[[14]](#endnote-14)^

Mucormycosis is characterised by the presence of hyphal invasion of sinus tissue and a time course of less than four weeks ^[[15]](#endnote-15)^, ^[[16]](#endnote-16)^ Clinically, rhinocerebral mucormycosis can present with atypical signs and symptoms, the initial symptoms of rhinocerebral mucormycosis are consistent with either sinusitis or periorbital cellulitis^[[17]](#endnote-17)^, ^[[18]](#endnote-18)^ and include eye or facial pain and facial numbness, followed by the onset of conjunctival suffusion, blurry vision, and soft tissue swelling^[[19]](#endnote-19)^ ^[[20]](#endnote-20)^ ^[[21]](#endnote-21)^. Fever is variable and may be absent in up to half of cases^[[22]](#endnote-22)^ . If untreated, infection usually spreads from the ethmoid sinus to the orbit, resulting in loss of extraocular muscle function and proptosis. Diplopia and marked chemosis may also be seen. The infection may rapidly extend into the neighboring tissues. On set of signs and symptoms in the contralateral eye, with resulting bilateral proptosis, chemosis, vision loss, and ophthalmoplegia, is an ominous sign that suggests the development of cavernous sinus thrombosis. Upon visual inspection, infected tissue may appear normal during the earliest stages of spread of the fungus. Infected tissue then progresses through an erythematous phase, with or without edema, before onset of a violaceous appearance, and finally the development of a black, necrotic eschar as the blood vessels become thrombosed and tissue infarction occurs^[[23]](#endnote-23)^ Infection can sometimes extend from the sinuses into the mouth causes loosening of teeth and often produce painful, necrotic ulcerations of the hard palate^[[24]](#endnote-24)^. Cranial nerve findings represent extensive infection and signal a grave prognosis. Progressive vision loss and ultimately blindness may result either from involvement of the optic nerve or from arteriolar invasion resulting in infarction^[[25]](#endnote-25)^ ^[[26]](#endnote-26)^ ^[[27]](#endnote-27)^ or from cavernous sinus thrombosis. Cranial nerves five and seven may also be affected, resulting in ipsilateral loss of facial sensation and ptosis and pupillary dilation ^[[28]](#endnote-28)^, ^[[29]](#endnote-29)^ Infection can also spread posteriorly from either the orbit or sinuses to the central nervous system. A bloody nasal discharge may be the first sign that infection has invaded through the terbinates and into the brain. When there is extensive central nervous system involvement, the angioinvasive nature of the fungus may result in cavernous sinus thrombosis and internal carotid artery encasement and thrombosis with extensive resulting cerebral infarctions^[[30]](#endnote-30)^ ^[[31]](#endnote-31)^ ^[[32]](#endnote-32)^. Occasionally cerebral vascular invasion may lead to hematogenous dissemination of the infection ^[[33]](#endnote-33)^ with or without development of mycotic aneurysms^[[34]](#endnote-34)^.

Pulmonary mucormycosis may develop as a result of inhalation or by hematogenous or lymphatic spread. Symptoms of pulmonary mucormycosis includes refractory fever on broad-spectrum antibiotics, non-productive cough, progressive dyspnea, pleuritic chest pain^[[35]](#endnote-35)^. High-resolution chest CT scan is the
best method of determining the extent of pulmonary mucormycosis and may demonstrate evidence of infection before it is seen on the chest x-ray^[[36]](#endnote-36)^.

Patients with skin barrier disruptions (burns, trauma, catheter insertion, injections) or persistent skin maceration are at increased risk for cutaneous mucormycosis ^[[37]](#endnote-37)^. The fungus can invade into adjacent fat, muscle, fascia, and even bone, while secondary vascular invasion and hematogenous spread are less common ^[[38]](#endnote-38)^ ^[[39]](#endnote-39)^ . However, cutaneous mucormycosis with hematogenous dissemination has high fatality rates ^[[40]](#endnote-40)^. **Gastrointestinal mucormycosis is** mainly occurs in patients who are extremely malnourished (especially infants or children) and is thought to arise from ingestion of the fungi. In particular, gastrointestinal mucormycosis has been seen in premature neonates, often in association with widespread disseminated disease.^[[41]](#endnote-41)^ ^[[42]](#endnote-42)^ ^[[43]](#endnote-43)^ ^[[44]](#endnote-44)^. The stomach, colon, and ileum are the most commonly involved sites. The stomach, colon, and ileum are the most commonly involved sites. The diagnosis may be made by biopsy of the suspected area during surgery or endoscopy.^[[45]](#endnote-45)^ .

Diagnosis of mucormycosis remains challenging. Clinical approach to diagnosis has a low sensitivity and specificity, it helps however in raising suspicion and prompting the initiation of laboratory testing. Histopathology, direct examination and culture remain essential tools, although the molecular methods are improving. Histological features include mycotic infiltration of blood vessels, vasculitis with thrombosis, tissue infarction, haemorrhage and acute neutrophilic infiltrate.^[[46]](#endnote-46)^^[[47]](#endnote-47)^, however, of a necrotic eschar does not preclude the diagnosis ^[[48]](#endnote-48)^ ^[[49]](#endnote-49)^. A definitive diagnosis is based on the demonstration of fungal hyphae typical for mucormycetes in biopsies of affected tissues, or bronchoalveolar lavage (BAL) in patients with pulmonary mucormycosis. Histopathology is a very important diagnostic tool since it distinguishes the presence of the fungus as a pathogen in the specimen from a culture contaminant and is indispensable to define whether there is blood vessel invasion.^[[50]](#endnote-50)^ Radiologically, multiple (≥10) nodules and pleural effusion are reportedly associated with pulmonary mucormycosis. Another finding on computerized tomography (CT) scan, which seems to indicate the presence of mucormycosis, is the reverse halo sign. Microscopy (direct and on histopathology) and culture are the cornerstones of diagnosis. Molecular assays can be used either for detection or identification of mucormycetes, and they can be recommended as valuable add-on tools that complement conventional diagnostic procedures.^[[51]](#endnote-51)^ MRI is a valuable modality that can be used to diagnose mucormycosis infections involving sino-nasal region, orbits, and possible intracranial extension. The multiplanar capabilities of MRI with its superior soft tissue depiction are helpful in delineating the anatomical extent of disease as well as its complications^[[52]](#endnote-52)^.

Another emerging imaging technique, which may eventually aid in the diagnosis and management of mucormycosis is the positron emission tomography-computed tomography (PET/CT) with [18F]- fluorodeoxyglucose (FDG).^[[53]](#endnote-53)^ When feasible, endobronchial ultrasound-guided fine needle aspiration is also a useful diagnostic tool.^[[54]](#endnote-54)^

The management of fungal infection includes administration of antifungal therapeutics with and without surgical interventions.^[[55]](#endnote-55)^ Surgery is necessary due to the massive amount of tissue necrosis occurring during mucormycosis, which may not be prevented by killing the organism.^[[56]](#endnote-56)^ In rhinocerebral mucormycosis, early surgical excision of the infected sinuses and appropriate debridement of the retroorbital space can often prevent the infection from extending into the eye^[[57]](#endnote-57)^, There is growing scientific reports on immune response of antifungal medication but still a large number of studies are not clinical based. Here, in case of fungal infection host response involves both cellular and humoral immunity. However, humoral immunity plays a larger role in offering protection against fungal infection. It has been also reported that cellular immunity associated with Th1-type offer a protectiveresponses against fungal infection via secreting IFN-y. On the contrary, the response of Th2 via IL4 and IL10 triggers and facilitate fungal infection and pathological outcomes. Further, mononuclear immune cells start infiltration at the site of infection and offering a delayed immune activity favoring fungal infections.^[[58]](#endnote-58)^ The poor immune surveillance and cellular immune activity was reported during chronic inflammatory diseases including diabetes and cancer.^[[59]](#endnote-59)^

The homoeopathy system includes a holistic approach, the medicines are selected on the basis of presenting signs and symptoms of each patient^[[60]](#endnote-60)^.Fungal infections are amenable to homoeopathic treatment. Various research studies undertaken on various fungi in-vitro model showed that homoeopathy medicine could prevent the growth of the fungus. prajapatiet al showed that homoeopathic medicines Syzygium jambolanum, Ficus religiosa, Ocimum sanctum, Allium cepa, Thuja occidentalis, Holarrhenaantidysenterica and Eucalyptus globulus showed signifi cant inhibitory activity against the growth of Candida. albicans^[[61]](#endnote-61)^, whereas gupta et al. study shown that homoeopathic medicines Mezereum in 200 and 30 potency showed maximum inhibition of growth of Candida albicans. ^[[62]](#endnote-62)^ ^[[63]](#endnote-63)^. Another invitro study demonstrated that homoeopathic drugs, namely Zingiber officinale, Holarrhenantidysenterica, Terminalia chebula, Allium cepa, Caesalpinia bonducella, Eucalyptus globulus, Ruta graveolens and Thuja occidentalis have significant antifungal activity against human pathogenic fungi Aspergillus niger.^[[64]](#endnote-64)^

Symptomatic Homoeopathy management of Suspected and Diagnosed cases of Mucormycosis has been advised in Guidelines by Ministry of AYUSH.^[[65]](#endnote-65)^ These guidelines will play a vital role in identification of the indicated medicines in this proposed study.

**Hypothesis**

Homoeopathic treatment, as an adjuvant to standard of care given to patients suffering from COVID-related mucormycosis, can lead to better recovery of the patients.

# OBJECTIVES:

## Primary Objective

- To evaluate the role of adjuvant homoeopathy in treatment of covid-related ROCM patients through standard parameters in respective conditions

## Secondary Objectives

- To enhance the survival of the patients hospitalized due to covid-related mucormycosis

# Material & Methods

## 3.1 STUDY DESIGN

Study design is single blind, randomized clinical trial.

Of the 2 groups running in parallel, the control arm will receive conventional treatment as per IMP, while the other group will receive homoeopathic intervention along with IMP. Allocation ratio will be 1:1 through randomisation obtained from computerised random number generator.

## 3.2 STUDY SETTING

**The trial will be conducted at** **St. George’s and JJ Hospital, Mumbai with informed consent of the patients admitted in the IPD for treatment of COVID related mucormycosis.**

## 3.3 ELIGIBILITY CRITERIA

### INCLUSION CRITERIA:

- Confirmed cases* of covid-related ROCM .
- Age > 18 years
- All genders
- ***Confirmation of each type of mucormycosis will be based upon** respective inclusion criteria:

| **S.No** | **Type of Mucormycosis** | **Inclusion criteria** |
| --- | --- | --- |
|  | **ROCM** | - Stage 1 and stage 2 (Staging document annexed) of ROCM will only be included). - Fungal smear analysis of the affected site reporting presence of mucormycetes (to save precious time), followed by MRI and/or biopsy - *MRI contrast imaging**   *MRI will show Mucosal thickening with T2 hypointense components at T2W images, Nonenhancement of involved mucosa/soft tissue at Post contrast T1W images, Marrow edema and enhancement of adjacent bones and skull base at Fat- saturated T2W and postcontrast T1W image. ^[[66]](#endnote-66)^ . Hyperintense lesion extending from paranasal sinus along orbital apex into intracranial structures and narrowing or slow flow in the ipsilateral internal carotid artery in the vicinity of mucor invasion seen in T2W^[[67]](#endnote-67)^.   - KOH staining/ microscopy and Biopsy**** of affected area   ****Biopsy will show non-septate/pauci-septate, ribbon-like hyphae (at least 6–16μm wide), Vessel occlusion)^[[68]](#endnote-68)^. |

### EXCLUSION CRITERIA:

- **ROCM:** Cases in stages 3 and 4(Staging document annexed) presenting with bony erosion, cerebral vascular invasion, invasion into the cranium, orbital apex or cribriform plate of the ethmoid bone, cavernous sinus (II-VI cranial nerve palsies) will be excluded.
- **Diagnosed cases of Pulmonary, Cutaneous, Gastrointestinal , Advanced disseminated mucormycosis will be excluded.**
- Pregnant and lactating women
- In the opinion of the clinical team, progression to death is imminent and inevitable within the next 24 hours, irrespective of the provision of treatments.

## 3.4 INTERVENTION

Through Patient Information Sheet, patient will be duly informed about equal chances of getting placebo or homoeopathic medicine, depending on the group s/he’s allotted, and then consent will be taken. Moreover, patients will be getting standard care of conventional medicine in both groups, and, therefore, would not be at risk.

### Details of intervention:

A detailed history and examination will be gathered from the already available information of the patient in the admission records at hospital. The patients randomised into experimental group will be interrogated by the homoeopathic physician only for the additional information required from Homoeopathy perspective.

As per each case, individualized homoeopathic medicine will be prescribed. Symptomatic Homoeopathy management of Suspected and Diagnosed cases of Mucormycosis has been advised in Guidelines by Ministry of AYUSH.^[[69]](#endnote-69)^ These guidelines will play a vital role in identification of the indicated medicines in this proposed study.

Daily recording of the administered dose will be recorded and also captured in the Case Record Form (CRF) developed by the Homoeopathy team, which will be having content other than, and in addition to the standard form, which will be filled at the hospital. If there is any change in dosage / prescription of medications during the study period, that will be mentioned with reason in the prescription chart. If these medicines are stopped, that too would be recorded with reason.

The patients assigned to the placebo group will be given placebo in the form of similar looking homoeopathic pills of 30 size, dispensed with the 30% V/V dispensing alcohol.

### Change of prescription

The prescribed medicine will be changed if no change in the next scheduled laboratory investigations or patient reports to be clinically unwell, even after at least 3 doses of the prescribed medicine.

**In either arm, no patient will be devoid of the conventional management as per IMP at any given point of time**. The allopathic medicines given during the study duration will be recorded specifically, with details of reason for administration, the name of the medicine with dose, along with any other relevant details, in the daily medication sheet as well as the CRF. The participants of the allopathy arm will get non-medicated, similar looking lactose globules, as placebos.

### Duration of intervention:

28 days. However, if patient is discharged before, the medicines will continue, if needed, and the follow up will be done telephonically on the 14^th^ and 28^th^ day of illness.

## 3.5 WITHDRAWAL OF A TRIAL PARTICIPANT:

- If the patient worsens, and mucormycosis expands beyond the range of inclusion criteria
- If the patient is in need of the ventilator support
- If the participant is unwilling to continue or turns non-compliant

## 3.6 OUTCOMES

#### Primary:

Change in condition as seen in standard imaging or other diagnostic techniques:

- MRI contrast in ROCM cases

#### Secondary:

- To assess difference in the survival of the patients in both the groups during hospitalization.

## LABORATORY INVESTIGATIONS

| **INVESTIGATIONS** | | |
| --- | --- | --- |
| - **Lab parameters** | CBC, ESR, FBS, PPBS, HbA1C, LFT, KFT with electrolytes | D0 and as per IMP |
| - **Imaging technique** | - CEMRI of Brain with orbits and sinuses. | - (Only at inclusion) |
|  | - CEMRI PNS | - D0 (or latest report if it is within 03 days of enrolment day), D14 and D28, or before or after as per IMP/ requirement |
|  | - CT guided endoscopy-cum-biopsy Fungal smear/KOH staining | - As per IMP, if required |

## TARGET SAMPLE SIZE:

Sample size was determined using the expected proportion of event / outcome in each group values of which are estimated from literature & using the formula,

n= (Z_α_ + Z_β_)^2^ [p q]

(p-q) ^2^

where Z_α_ is the z variate of alpha error i.e. a constant with value 1.96

Z_β_ i.e. a constant with value 0.84

p, q are proportions of the variable, values of which are taken from the parent article.

(Chow S, Shao J, Wang H. 2008. Sample Size Calculations in Clinical Research. 2nd Ed. Chapman & Hall/CRC Biostatistics Series. **page 89**.)

Approximate estimates:

1. 80% power
2. Type I error to be 5%
3. Type II error to be 20%
4. Difference in proportions between the groups to be 0.10

Substituting the values,

n= (1.96+ 0.84)^2^ [0.09)]

(0.10)^2^

n= 70.56

A minimum of **71 subjects per group** completing the study would be giving a good external validity. Since the number has to be equally divided in both the groups, **72 participants will be enrolled.**

For follow-up studies, to avoid loss by loss to follow up / attrition, kindly consider recruiting 5-25% more subjects so that even after attrition, we would be able to achieve the required minimum sample size.

**Statistical analysis:**

Data collected will be compiled on to a MS Office excel worksheet & will be subjected to statistical analysis using an appropriate package like SPSS software. Descriptive statistics like frequency (n) & percentage (%) of categorical data, mean & Standard deviation of numerical data in each group / subgroup will be depicted.

Frequency (n) & percentage (%) of various categories in each group / subgroup will be compared using chi square test.

Normality of numerical data will be checked using Shapiro – Wilk test or Kolmogorov-Smirnov test. Depending on the normality of data, statistical tests will be determined.

For a numerical continuous data following a normal distribution, inter group comparison (2 groups) will be done using t test, else a non parametric substitute like Mann Whitney U test will be used.

Intra group comparisons for a numerical continuous data following a normal distribution will be done using paired t test (for 2 observations) or repeated measures ANOVA for >2 observations, else a non parametric substitute like Wilcoxon signed rank test (for 2 observations) or Friedman’s test for >2 observations will be used.

Frequency (n) & percentage (%) of various responses in each time interval will be compared using chi square test / McNemar’s test.

Keeping alpha error at 5% and Beta error at 20%, power at 80%, p<0.05 will be considered statistically significant.

## PHASE OF TRIAL:

Phase II

## ESTIMATED DURATION OF TRIAL:

3 months

## DURATION OF ADMINISTRATION

30 days

## DATA COLLECTION METHODS

Considering the critical importance of COVID related ROCM , data from the trial participants will be collected extensively. All the below mentioned documents will be developed and retained.

- CRF of covid related ROCM.
- A database that stores the above information for each patient with the subsequent observations by the physician at every visit
- Reports of all laboratory investigations to help physicians understand efficacy of diagnostic tools and/or interventions.
- All data pertaining to homoeopathy, like case taking, repertorisation chart and prescription decisions will be maintained.

## STATISTICAL METHODS

Data analysis will be done through Intention To Treat (ITT) method, thus, all randomized patients should be included in the primary analysis. Additional analyses (e.g. subgroup analysis as per age, sex, perceived risk categories will also be carried out. Statistical advice will be sought before initiation. Kolmogorov-Smirnov tests will be used to analyse the normality of the data distribution. Data will be presented in number, percentage, Mean with 95% Confidence Interval, SD, Median, IQR. Statistical significance will be considered as the p value<0.05. Correlations between individual diagnoses/symptoms/ characteristics will be calculated in a Spearman rank correlation table. Correlations between groups of symptoms will be explored by principal component analysis (PCA). Comparison of baseline characteristics and outcome among all participants will be done by using parametric or non-parametric test. For categorical data χ2 exact test will be used. Concordance of the different methods for appraising result will be measured by Cohen’s kappa and Spearman rank correlation.

# ETHICS AND DISSEMINATION

## Research ethics approval

The trial will initiate after due approval of the Institutional Ethics committee (EC) of St. George’s and JJ Group of hospitals and CCRH.

## Insurance

CCRH will provide compensation and insurance cover in the trial for the said duration of study, according to the terms finalized under clinical trial cover with the identified insurance firm.

## Consent

Individuals above the age of 18 years will be considered eligible for consent, and informed, electronic (video) consent will be taken, in presence of a witness.

## Confidentiality

Confidentiality of the participants will be maintained, and data provided used only for research and learning purposes.

## Access to data

Since the COVID-19 is sensitive issue from various aspects, the research team and funding agency will only have access to the data and final trial dataset.

## Dissemination policy

The Investigators will communicate trial results to participants, healthcare professionals, the public, and other relevant groups (via publication, reporting in results databases, or other data sharing arrangements) after completion of the study. However, no information based on unjustified claims, or the findings of interim analysis would be communicated in any form.

## Publication Details:

Will be published in a reputed, peer-reviewed medical journal.

## CTRI registration:

The trial will be registered with Clinical Study Registry of India (CTRI) after EC approval and before recruitment of the participant begins.

# References:

1. Singh AK, Singh R, Joshi SR, Misra A, Mucormycosis in COVID-19: Asystematic review of cases reported worldwide and in India, Diabetes & Metabolic Syndrome: ClinicalResearch& Reviews (2021), doi: https://doi.org/10.1016/j.dsx.2021.05.019. [↑](#endnote-ref-1)
2. Kumar G, Adams A, Hererra M, Rojas ER, Singh V, Sakhuja A, et al. Predictors and outcomes of hais in COVID- 19 patients. Int J Infect Dis. 2020;104(3):287–92. [↑](#endnote-ref-2)
3. 5. Kimmig LM, Wu D, Gold M, Pettit NN, Pitrak D, Mueller J, et al. IL-6 inhibition in critically Ill COVID-19 patients is associated with increased secondary infections. Front Med (Lausanne). 2020;7:583897. [↑](#endnote-ref-3)
4. Garg D, Muthu V, Sehgal IS, Ramachandran R, Kaur H, Bhalla A, Puri GD, Chakrabarti A, Agarwal R. Coronavirus Disease (Covid-19) Associated Mucormycosis (CAM): Case Report and Systematic Review of Literature. Mycopathologia. 2021 May;186(2):289-298. doi: 10.1007/s11046-021-00528-2. Epub 2021 Feb 5. PMID: 33544266; PMCID: PMC7862973. [↑](#endnote-ref-4)
5. Richardson, M. The ecology of the Zygomycetes and its impact on environmental exposure. Clin. Microbiol.

   Infect. 2009, 15, 2–9. [↑](#endnote-ref-5)
6. 2. Roden, M.M.; Zaoutis, T.E.; Buchanan,W.L.; Knudsen, T.A.; Sarkisova, T.A.; Schaufele, R.L.; Sein, M.; Sein, T.; Chiou, C.C.; Chu, J.H.; et al. Epidemiology and outcome of zygomycosis: A review of 929 reported cases. Clin. Infect. Dis. 2005, 41, 634–653. [↑](#endnote-ref-6)
7. Sugar AM. In: Mandell GL, Bennett JE, Dolin R(eds) Mandell, Douglas, and Bennett’s principles and practice of infectious diseases (5th edn), Churchill Livingstone, New York, USA, 2000. [↑](#endnote-ref-7)
8. Song G, Liang G, Liu W. Fungal Co-infections Associated with Global COVID19 Pandemic: A Clinical and Diagnostic Perspective from China. Mycopathologia. 2020 Aug;185(4):599-606. [↑](#endnote-ref-8)
9. Singh AK, Singh R, Joshi SR, Misra A, Mucormycosis in COVID-19: Asystematic review of cases reported worldwide and in India, Diabetes & Metabolic Syndrome: ClinicalResearch& Reviews (2021), doi: https://doi.org/10.1016/j.dsx.2021.05.019. [↑](#endnote-ref-9)
10. Müller, J.A., Groß, R., Conzelmann, C. et al. SARS-CoV-2 infects and replicates in cells of the human endocrine and exocrine pancreas. Nat Metab 3, 149–165 (2021). https://doi.org/10.1038/s42255-021-00347-1 [↑](#endnote-ref-10)
11. ang X, Uhl S, Zhang T, Xue D, Li B, Vandana JJ, Acklin JA, Bonnycastle LL, Narisu N, Erdos MR, Bram Y, Chandar V, Chong ACN, Lacko LA, Min Z, Lim JK, Borczuk AC, Xiang J, Naji A, Collins FS, Evans T, Liu C, tenOever BR, Schwartz RE, Chen S. SARS-CoV-2 infection induces beta cell transdifferentiation. Cell Metab. 2021 May 19:S1550-4131(21)00232-1. doi: 10.1016/j.cmet.2021.05.015. Epub ahead of print. PMID: 34081913; PMCID: PMC8133495. [↑](#endnote-ref-11)
12. Singh AK, Singh R, Joshi SR, Misra A, Mucormycosis in COVID-19: Asystematic review of cases reported worldwide and in India, Diabetes & Metabolic Syndrome: ClinicalResearch& Reviews (2021), doi: https://doi.org/10.1016/j.dsx.2021.05.019. [↑](#endnote-ref-12)
13. Singh AK, Singh R, Joshi SR, Misra A, Mucormycosis in COVID-19: Asystematic review of cases reported worldwide and in India, Diabetes & Metabolic Syndrome: ClinicalResearch& Reviews (2021), doi: https://doi.org/10.1016/j.dsx.2021.05.019. [↑](#endnote-ref-13)
14. Hartnett KP, Jackson BR, Perkins KM, Glowicz J, Kerins JL, Black SR, Lockhart SR, Christensen BE, Beer KD. A Guide to Investigating Suspected Outbreaks of Mucormycosis in Healthcare. J Fungi (Basel). 2019 Jul 24;5(3):69. doi: 10.3390/jof5030069. PMID: 31344775; PMCID: PMC6787571. [↑](#endnote-ref-14)
15. Ferguson BJ. Definitions of fungal rhinosinusitis. Otolaryngol Clin North Am 2000;33:227–35 [↑](#endnote-ref-15)
16. Chakrabarti A, Denning DW, Ferguson BJ, Ponikau J, Buzina W, Kita H et al. Fungal rhinosinusitis: a categorization and definitional schema addressing current controversies. Laryngoscope 2009;119:1809–18 [↑](#endnote-ref-16)
17. Dhiwakar, M., A. Thakar, and S. Bahadur. 2003. Improving outcomes in rhinocerebral mucormycosis–early diagnostic pointers and prognostic factors. J. Laryngol Otol. 117:861–865. [↑](#endnote-ref-17)
18. Talmi, Y. P., A. Goldschmied-Reouven, M. Bakon, I. Barshack, M. Wolf, Z. Horowitz, M. Berkowicz, N. Keller, and J. Kronenberg. 2002. Rhino-orbital and rhino-orbito-cerebral mucormycosis. Otolaryngol. Head Neck Surg. 127:22–31 [↑](#endnote-ref-18)
19. Khor, B. S., M. H. Lee, H. S. Leu, and J. W. Liu. 2003. Rhinocerebral mucormycosis in Taiwan. J. Microbiol. Immunol. Infect. 36:266–269 [↑](#endnote-ref-19)
20. Peterson, K. L., M. Wang, R. F. Canalis, and E. Abemayor. 1997. Rhinocerebral mucormycosis: evolution of the disease and treatment options. Laryngoscope 107:855–862. [↑](#endnote-ref-20)
21. Peterson, K. L., M. Wang, R. F. Canalis, and E. Abemayor. 1997. Rhinocerebral mucormycosis: evolution of the disease and treatment options. Laryngoscope 107:855–862. [↑](#endnote-ref-21)
22. Talmi, Y. P., A. Goldschmied-Reouven, M. Bakon, I. Barshack, M. Wolf, Z. Horowitz, M. Berkowicz, N. Keller, and J. Kronenberg. 2002. Rhino-orbital and rhino-orbito-cerebral mucormycosis. Otolaryngol. Head Neck Surg. 127:22–31. [↑](#endnote-ref-22)
23. Husain, S., B. D. Alexander, P. Munoz, R. K. Avery, S. Houston, T. Pruett, R. Jacobs, E. A. Dominguez, J. G. Tollemar, K. Baumgarten, C. M. Yu, M. M. Wagener, P. Linden, S. Kusne, and N. Singh. 2003. Opportunistic mycelial fungal infections in organ transplant recipients: emerging importance of non-Aspergillus mycelial fungi. Clin. Infect. Dis. 37:221–229 [↑](#endnote-ref-23)
24. Petrikkos, G., A. Skiada, H. Sambatakou, A. Toskas, G. Vaiopoulos, M. Giannopoulou, and N. Katsilambros. 2003. Mucormycosis: ten-year experience at a tertiary-care center in Greece. Eur. J. Clin. Microbiol. Infect. Dis. 22:753–756. [↑](#endnote-ref-24)
25. Hussain, S., N. Salahuddin, I. Ahmad, I. Salahuddin, and R. Jooma. 1995. Rhinocerebral invasive mycosis: occurrence in immunocompetent individuals. Eur. J. Radiol. 20:151–155. [↑](#endnote-ref-25)
26. Thajeb, P., T. Thajeb, and D. Dai. 2004. Fatal strokes in patients with rhino-orbito-cerebral mucormycosis and associated vasculopathy. Scand. J. Infect. Dis. 36:643–648 [↑](#endnote-ref-26)
27. Sponsler, T. A., J. W. Sassani, L. N. Johnson, and J. Towfighi. 1992. Ocular invasion in mucormycosis. Survey Ophthalmol. 36:345–350 [↑](#endnote-ref-27)
28. Dhiwakar, M., A. Thakar, and S. Bahadur. 2003. Improving outcomes in rhinocerebral mucormycosis–early diagnostic pointers and prognostic factors. J. Laryngol Otol. 117:861–865 [↑](#endnote-ref-28)
29. Peterson, K. L., M. Wang, R. F. Canalis, and E. Abemayor. 1997. Rhinocerebral mucormycosis: evolution of the disease and treatment options. Laryngoscope 107:855–862. [↑](#endnote-ref-29)
30. Anaissie, E. J., and A. H. Shikhani. 1985. Rhinocerebral mucormycosis with internal carotid occlusion: report of two cases and review of the literature. Laryngoscope 95:1107–1113 [↑](#endnote-ref-30)
31. Anaissie, E. J., and A. H. Shikhani. 1985. Rhinocerebral mucormycosis with internal carotid occlusion: report of two cases and review of the literature. Laryngoscope 95:1107–1113 [↑](#endnote-ref-31)
32. Thajeb, P., T. Thajeb, and D. Dai. 2004. Fatal strokes in patients with rhino-orbito-cerebral mucormycosis and associated vasculopathy. Scand. J. Infect. Dis. 36:643–648 [↑](#endnote-ref-32)
33. Pillsbury, H. C., and N. D. Fischer. 1977. Rhinocerebral mucormycosis. Arch. Otolaryngol. 103:600–604. [↑](#endnote-ref-33)
34. Sehgal, A., M. Raghavendran, D. Kumar, A. Srivastava, D. Dubey, and A. Kumar. 2004. Rhinocerebral mucormycosis causing basilar artery aneurysm with concomitant fungal colonic perforation in renal allograft recipient: a case report. Transplantation 78:949–950. [↑](#endnote-ref-34)
35. Management protocol for mucormycosis. All India institute of Medical Sciences, Rishikesh, Uttarakhand. Available at https://aiimsrishikesh.edu.in/a1_1/wp-content/uploads/2020/11/MUCOR-Management-protocol_AIIMS-Rishikesh_Version-1.0.cleaned.pdf. [↑](#endnote-ref-35)
36. Spellberg B, Edwards J Jr, Ibrahim A. Novel perspectives on mucormycosis: pathophysiology, presentation, and management. Clin Microbiol Rev. 2005 Jul;18(3):556-69. doi: 10.1128/CMR.18.3.556-569.2005. PMID: 16020690; PMCID: PMC1195964. [↑](#endnote-ref-36)
37. Spellberg B, Edwards Jr J, Ibrahim A. Novel perspectives on mucormycosis: pathophysiology, presentation, and management. Clin Microbiol Rev 2005;18(3):556–69 [↑](#endnote-ref-37)
38. Roden MM, Zaoutis TE, Buchanan WL, et al. Epidemiology and outcome of zygomycosis: a review of 929 reported cases. Clin Infect Dis 2005;41:634–53 [↑](#endnote-ref-38)
39. Petrikkos G, Skiada A, Lortholary O, Roilides E, Walsh TJ, Kontoyiannis DP. Epidemiology and clinical manifestations of mucormycosis. Clin Infect Dis 2012;54(1):S23–34. [↑](#endnote-ref-39)
40. Roden MM, Zaoutis TE, Buchanan WL, et al. Epidemiology and outcome of zygomycosis: a review of 929 reported cases. Clin Infect Dis 2005;41:634–53 [↑](#endnote-ref-40)
41. min, S. B., R. M. Ryan, L. A. Metlay, and W. J. Watson. 1998. Absidia corymbifera infections in neonates. Clin. Infect. Dis. 26:990–992 [↑](#endnote-ref-41)
42. Craig, N. M., F. L. Lueder, J. M. Pensler, B. S. Bean, M. L. Petrick, R. B. Thompson, and L. R. Eramo. 1994. Disseminated Rhizopus infection in a premature infant. Pediatr. Dermatol. 11:346–350 [↑](#endnote-ref-42)
43. Kecskes, S., G. Reynolds, and G. Bennett. 1997. Survival after gastrointestinal mucormycosis in a neonate. J. Paediatr. Child Health 33:356–359 [↑](#endnote-ref-43)
44. Sharma, M. C., S. S. Gill, S. Kashyap, R. Kataria, D. K. Gupta, P. Sahni, and S. K. Acharya. 1998. Gastrointestinal mucormycosis–an uncommon isolated mucormycosis. Indian J. Gastroenterol. 17:131–133. [↑](#endnote-ref-44)
45. Spellberg B, Edwards J Jr, Ibrahim A. Novel perspectives on mucormycosis: pathophysiology, presentation, and management. Clin Microbiol Rev. 2005 Jul;18(3):556-69. doi: 10.1128/CMR.18.3.556-569.2005. PMID: 16020690; PMCID: PMC1195964. [↑](#endnote-ref-45)
46. DeShazo RD, Chapin K, Swain RE. Fungal sinusitis. N Engl J Med 1997;337:254–9 [↑](#endnote-ref-46)
47. Sharma S, Grover M, Bhargava S, Samdani S, Kataria T. Post coronavirus disease mucormycosis: a deadly addition to the pandemic spectrum. J Laryngol Otol. 2021 Apr 8:1-6. doi: 10.1017/S0022215121000992. Epub ahead of print. PMID: 33827722; PMCID: PMC8060545. [↑](#endnote-ref-47)
48. Mohindra S, Mohindra S, Gupta R, Bakshi J, Gupta SK. Rhinocerebralmucormycosis: the disease spectrum in 27 patients. Mycoses 2007;50:290–6 [↑](#endnote-ref-48)
49. Munir N, Jones NS. Rhinocerebralmucormycosis with orbital and intracranial extension: a case report and review of optimum management. JLaryngolOtol2007;121:192–5 [↑](#endnote-ref-49)
50. Guarner, J.; Brandt, M.E. Histopathologic Diagnosis of Fungal Infections in the 21st Century. *Clin. Microbiol. Rev.*2011, *24*, 247–280. [↑](#endnote-ref-50)
51. Skiada A, Lass-Floerl C, Klimko N, Ibrahim A, Roilides E, Petrikkos G. Challenges in the diagnosis and treatment of mucormycosis. Med Mycol. 2018 Apr 1;56(suppl_1):93-101. doi: 10.1093/mmy/myx101. PMID: 29538730; PMCID: PMC6251532. [↑](#endnote-ref-51)
52. Awal, S.S., Biswas, S.S. & Awal, S.K. Rhino-orbital mucormycosis in COVID-19 patients—a new threat?. *Egypt J Radiol Nucl Med* **52,**152 (2021). https://doi.org/10.1186/s43055-021-00535-9 [↑](#endnote-ref-52)
53. Liu Y, Wu H, Huang F, Fan Z, Xu B. Utility of 18F- FDG PET/CT in diagnosis and management of mucormycosis. Clin Nucl Med. 2013; 38: e370–e371. [↑](#endnote-ref-53)
54. 29. Nair V, Sharma RK, Khanna A, Talwar D. Pulmonary mucormycosis diagnosed by convex probe endobronchial ultrasound-guided fine needle aspiration of cavity wall. Lung India. 2017; 34: 179–181 [↑](#endnote-ref-54)
55. Skiada A, Lass-Floerl C, Klimko N, Ibrahim A, Roilides E, Petrikkos G, et al. Challenges in the diagnosis and treatment of mucormycosis. Med Mycol. 2018;56(1):S93–S101. doi:10.1093/mmy/myx101. [↑](#endnote-ref-55)
56. Ibrahim,A.S., B. Spellberg, V. Avanessian, Y. Fu, and J. E. Edwards. 2005 *Rhizopus oryzae* adheres to, is phagocytosed by, and damages endothelial cells in vitro. Infect. Immun. 73:778–783 [↑](#endnote-ref-56)
57. Nithyanandam, S., M. S. Jacob, R. R. Battu, R. K. Thomas, M. A. Correa, and O. D’Souza. 2003. Rhino-orbito-cerebral mucormycosis. A retrospective analysis of clinical features and treatment outcomes. Indian J. Ophthalmol. 51:231–236 [↑](#endnote-ref-57)
58. Verma A, Wuthrich M, Deepe G, Klein B. Adaptive Immunity to Fungi. Cold Spring Harbor Perspect Med. 2015;5:a019612. doi:10.1101/cshperspect.a019612 [↑](#endnote-ref-58)
59. Prakash H, Ghosh AK, Rudramurthy SM, Singh P, Xess I, Savio J, et al. A prospective multicenter study on mucormycosis in India: Epidemiology, diagnosis, and treatment. Med Mycol. 2019;57(4):395– 402. doi:10.1093/mmy/myy060. [↑](#endnote-ref-59)
60. Guidelines for Homoeopathic practitioners for COVID-19; available at https://www.ayush.gov.in/docs/homeopathy-guidelines.pdf [↑](#endnote-ref-60)
61. Prajapati S, Sharma M, Gupta P, Kumar M, Dwivedi B, Arya BS. Evaluation of antifungal activity of different homoeopathic mother tinctures against Candida albicans. Indian J Res Homoeopathy 2017;11:237-43. [↑](#endnote-ref-61)
62. Gupta G, Garg K.L., Chandra B. Effect of homoeopathic drugs against fungi isolated fromhuman patients. Asian Homoeopathic Journal. 1995; 5(1): 15-18. [↑](#endnote-ref-62)
63. Gupta G, Srivastava AK, Gupta N, Gupta G, Mishra S. Anti-candidal activity of homoeopathic drugs: An in-vitro evaluation. Indian J Res Homoeopathy 2015;9:79-85. [↑](#endnote-ref-63)
64. Prajapati S, Sharma M, Kumar A, Gupta P, Dwivedi B, Arya BS, et al. Antimicrobial activity of different homoeopathic drugs and their potencies against ‘Aspergillus niger’ In vitro. Indian J Res Homoeopathy 2019;13:150-8. [↑](#endnote-ref-64)
65. Information for Homoeopathy Practitioners for Symptomatic Management of Suspected and Diagnosed cases of Mucormycosis. Available at https://www.ayush.gov.in/docs/Information%20for%20homoeopathic%20management%20of%20Muc rmycosis-reg.pdf [↑](#endnote-ref-65)
66. Sreshta K, Dave TV, Varma DR, Nair AG, Bothra N, Naik MN, Sistla SK. Magnetic resonance imaging in rhino-orbital-cerebral mucormycosis. Indian J Ophthalmol. 2021 Jul;69(7):1915-1927. doi: 10.4103/ijo.IJO_1439_21. PMID: 34146057. [↑](#endnote-ref-66)
67. Lone Parveen A, Wani Nisar A, Jehangir Majid. Rhino-orbito-cerebral mucormycosis: Magnetic resonance imaging. Indian Journal of Otology.2015.21(3). 215-218. [↑](#endnote-ref-67)
68. Management protocol for mucormycosis. All India institute of Medical Sciences, Rishikesh, Uttarakhand. Available at https://aiimsrishikesh.edu.in/a1_1/wp-content/uploads/2020/11/MUCOR-Management-protocol_AIIMS-Rishikesh_Version-1.0.cleaned.pdf. [↑](#endnote-ref-68)
69. Information for Homoeopathy Practitioners for Symptomatic Management of Suspected and Diagnosed cases of Mucormycosis. Available at https://www.ayush.gov.in/docs/Information%20for%20homoeopathic%20management%20of%20Muc rmycosis-reg.pdf

    **Annexure 1**

    **Staging of Rhino-Orbito-Cerebral Mucormycosis (ROCM)**

    Source: Honavar SG. Code Mucor: Guidelines for the Diagnosis, Staging and Management of Rhino-Orbito-Cerebral Mucormycosis in the Setting of COVID-19. Indian J Ophthalmol. 2021 Jun;69(6):1361-1365. Doi: 10.4103/ijo.IJO_1165_21. PMID: 34011699.

    | ***Staging of Rhino-orbito-Cerebral Mucomycosis*** | ***Symptoms*** | ***Signs*** |
    | --- | --- | --- |
    | **Stage:1 Involvement of the Nasal mucosa**  **1a**: Limited to the middle turbinate  **1b**: involvement of the inferior turbinate or ostium of the nasolaciacrimal duct  **1c**: involvement of the nasal septum  **1d**: Bilateral nasal mucosal involvement | Nasal Stuffiness, nasal discharge, foul smell, epistaxis | Foul-smelling sticky mucoid or haemorrhagic nasal discharge, nasal mucosal, inflammation, erythema, Violaceous or blue discoloration, pale ulcer, anaesthesia, ischemia, eschar |
    | **Stage:2 Involvement of Paranasal Sinuses**  **2a**: One sinus  **2b**: Two ipsilateral sinuses  **3c**: >Two ipsilateral sinuses and/or palate/oral cavity  **3d**: Bilateral paranasal sinus involvement or involvement of the zygoma or mandible | Symptoms in stage 1+ facial pain, facial edema, dental pain, systemic symptoms (malaise fever) | Signs stage 1+ unilateral or bilateral localized or diffuse facial edema, edema localized over the sinuses, localized sinus tenderness |
    | **Stage:3 Involvement of the Orbit**  **3a**: Nasolacrimal duct, medial orbit, vision unaffected  **3b**: Diffuse orbital involvement (.1 quadrant or >2 structures), vision unaffected  **3c**: Central retinal artery or ophthalmic artery occlusion or superior ophthalmic vein thrombosis; involvement of the superior orbital fissure, inferior orbital fissure, orbital apex, loss of vision | Symptoms in stages 1 and 2 + pain in the eyes, proptosis, ptosis, diplopia, loss of vision, infraorbital and facial V1 V2 nerve anesthesia | Signs in stages 1 and 2+ conjunctival chemoses, isolated ocular motility restriction ptosis, Proptosis, infraorbital nerve anesthesia, central retinal artery occlusion, features of ophthalmic vein thrombosis. V1 and V2 nerve anesthesia, and features of III , IV and VI nerve palsy indicating orbital apex/superior orbital fissure involvement. |
    | **Stage:4 Involvement of the CNS**  **4a:** Focal or partial cavernous sinus involvement and/or involvement of the cribriform plate  **4b:** Diffuse cavernous sinus involvement and/or cavernous sinus thrombosis  **4c:** Involvement beyond the cavernous sinus, involvement of the skull base, internal carotid artery occlusion, brain infraction  **4d:** multifocal or diffuse CNS disease | Symptoms stages 1 and 3+ bilateral proptosis, paralysis, altered consciousness, focal seizures | Signs in Stages 1-3 (same features overlap with Stage 3) + V1 and V2 nerve anesthesia ptosis, and features of III, IV and VI nerve palsy indicate cavernous sinus involvement. Bilaterally of these signs with contralateral orbital edema with no clinic-radiological evidence of paranasal sinus or orbital involvement on the contralateral side indicate cavernous sinus thrombosis. Hemiparesis, altered consciousness and focal seizure indicate brain invasion and infraction. |

    [↑](#endnote-ref-69)
